# Macrophage targeted theranostic strategy for accurate detection and rapid stabilization of the inflamed high-risk plaque

**DOI:** 10.7150/thno.59759

**Published:** 2021-08-18

**Authors:** Joon Woo Song, Hyeong Soo Nam, Jae Won Ahn, Hyun-Sang Park, Dong Oh Kang, Hyun Jung Kim, Yeon Hoon Kim, Jeongmoo Han, Jah Yeon Choi, Seung-Yul Lee, Sunwon Kim, Wang-Yuhl Oh, Hongki Yoo, Kyeongsoon Park, Jin Won Kim

**Affiliations:** 1 Multimodal Imaging and Theranostic Lab., Cardiovascular Center, Korea University Guro Hospital, Seoul, South Korea.; 2 Department of Mechanical Engineering, Korea Advanced Institute of Science and Technology, Daejeon, South Korea.; 3 Department of Systems Biotechnology, Chung-Ang University, Anseong, South Korea.

**Keywords:** atherosclerosis, targeted theranostics, drug delivery, PPARγ, OCT-NIRF

## Abstract

**Rationale:** Inflammation plays a pivotal role in the pathogenesis of the acute coronary syndrome. Detecting plaques with high inflammatory activity and specifically treating those lesions can be crucial to prevent life-threatening cardiovascular events.

**Methods:** Here, we developed a macrophage mannose receptor (MMR)-targeted theranostic nanodrug (mannose-polyethylene glycol-glycol chitosan-deoxycholic acid-cyanine 7-lobeglitazone; MMR-Lobe-Cy) designed to identify inflammatory activity as well as to deliver peroxisome proliferator-activated gamma (PPARγ) agonist, lobeglitazone, specifically to high-risk plaques based on the high mannose receptor specificity. The MMR-Lobe-Cy was intravenously injected into balloon-injured atheromatous rabbits and serial *in vivo* optical coherence tomography (OCT)-near-infrared fluorescence (NIRF) structural-molecular imaging was performed.

**Results:** One week after MMR-Lobe-Cy administration, the inflammatory NIRF signals in the plaques notably decreased compared to the baseline whereas the signals in saline controls even increased over time. In accordance with *in vivo* imaging findings, *ex vivo* NIRF signals on fluorescence reflectance imaging (FRI) and plaque inflammation by immunostainings significantly decreased compared to oral lobeglitazone group or saline controls. The anti-inflammatory effect of MMR-Lobe-Cy was mediated by inhibition of TLR4/NF-κB pathway. Furthermore, acute resolution of inflammation altered the inflamed plaque into a stable phenotype with less macrophages and collagen-rich matrix.

**Conclusion:** Macrophage targeted PPARγ activator labeled with NIRF rapidly stabilized the inflamed plaques in coronary sized artery, which could be quantitatively assessed using intravascular OCT-NIRF imaging. This novel theranostic approach provides a promising theranostic strategy for high-risk coronary plaques.

## Introduction

Inflammation promotes the rupture of fibrous cap within an atherosclerotic plaque and precipitates a fatal acute myocardial infarction 1. While statin is well-known to provide clinical benefits in numerous trials including optical coherence tomography (OCT) 2, 3, intravascular ultrasound 4, 5, and PET-CT 6, data from clinical studies regarding stabilizing effects of statin on the high-risk plaques in acute phase have been still inconclusive 7-9. Nonetheless, based on the growing evidences supporting a key role of inflammation in acute coronary events 10-13, acutely administered anti-inflammatory therapy seems to be beneficial by rapidly stabilizing the atheroma.

Peroxisome proliferator-activated receptor gamma (PPARγ) agonist is known to have pleiotropic anti-inflammatory and anti-atherosclerotic efficacy 14. However, as a higher dose of PPARγ agonist to enhance sufficient therapeutic effects on the plaque is associated with systemic adverse effects 14, targeted drug delivery is emerging as a promising strategy to increase the local drug concentration at the lesions and to avoid undesirable systemic effects 15-21. Our group previously developed a MMR targeted PPARγ agonist, lobeglitazone, delivery system (MMR-Lobe). While MMR-Lobe effectively reduced plaque burden and inflammation *via* activation of cholesterol efflux in macrophage foam cells 22, a long-term administration of MMR-Lobe should be required to fully enhance the anti-atherosclerotic effects on the murine atheroma. Until now, current treatments are ineffective to sufficiently stabilize the inflamed plaque in acute phase of coronary artery disease. Given the intravenous route of MMR-Lobe administration, inevitable multiple injection requirement is a critical hurdle for clinical application. Furthermore, the efficacy proved only in murine model limited the potential for translational application in the coronary plaque and an injectable form of the targeted strategy needs to be simultaneously applicable for both therapy and diagnosis.

To address these unmet needs, here, we developed a novel macrophage-targeted NIRF-emitting PPARγ activator, which integrated both diagnostic and therapeutic abilities in one agent, and applied this theranostic agent in rabbit models for both imaging and treatment of coronary-sized inflamed plaques, particularly in acute settings. We scaled up the MMR binding drug carrier, labeled it with near-infrared fluorescence dye (Cy7), and then incorporated PPARγ agonist, lobeglitazone, into it (MMR-Lobe-Cy). Combining MMR-Lobe-Cy with the OCT-NIRF structural-molecular imaging, which has the ability to simultaneously detect the inflammatory activity and plaque structure in coronary arteries 23-27, allows us to quantitatively estimate the specific delivery of loaded PPARγ agonist to plaque macrophages and serially assess dynamic changes of plaque inflammatory activity. We evaluated whether this novel strategy of OCT-NIRF imaging with MMR-Lobe-Cy could accurately localize inflamed high-risk atheroma and rapidly suppress the inflammation in coronary-sized atheroma. Additionally, we provided the mechanistic evidence for our strategy and explored whether the anti-inflammatory activity following a short-term treatment of MMR-Lobe-Cy could alter the plaque characteristics with respect to transition of atheroma composition into a more stable phenotype.

## Methods

### Materials

Maleimide-polyethylene glycol 2000-succinimidyl carboxymethyl ester (MAL-PEG-NHS) was obtained from JenKem Technology (Plano, TX, USA). Glycol chitosan (GC), deoxycholic acid (DOCA), mannosamine hydrochloride (MAN), trimethylamine (TEA), dimethylformamide (DMF), lipopolysaccharide (LPS), mannan, dimethyl sulfoxide (DMSO), 1-ethyl-3-(3-dimethylaminopropyl)-carbodiimide hydrochloride (EDC), N-hydroxysuccinimide (NHS), methanol (MeOH), ethanol (EtOH), DMSO-d_6_, deuterium oxide (D_2_O), chloroform-d, and low-density lipoprotein (LDL) were purchased from Sigma-Aldrich (St. Louis, MO, USA). Cy7-NHS ester was ordered from Lumiprobe Corporation (Hunt Valley, MD, USA). Lobeglitazone (CDK-501) was obtained from Chong Kun Dang Pharmaceutical Corp. (Seoul, South Korea) and dialysis membranes (molecular weight cut-off (MWCO): 1, 6-8, and 12-14 kDa) were purchased from Spectrum Laboratories, Inc. (Rancho Dominguez, CA, USA).

### Synthesis and characterizations of MMR-Lobe-Cy

GC was dissolved in the co-solvent of deionized water:MeOH (1:2, v/v) and reacted with pre-activated DOCA for 12 h. Then, the pre-activated MAN-PEG-NAC in the presence of EDC and NHS in MeOH was added to the solution and reacted for 12 h to fabricate the MAN-PEG-GC-DOCA (termed as MMR carrier). The mixture was then dialyzed against deionized water (DW) containing 30% EtOH for 2 days and DW for additional 2 days using a dialysis membrane (MWCO: 12-14 kDa), and then lyophilized to yield the MMR carrier. The synthesis of the MMR carrier was analyzed with a ^1^H-nuclear magnetic resonance (NMR) spectrometer (600 MHz NMR; Varian, Palo Alto, CA, USA). To synthesize a targetable imaging probe, MMR-Cy, the MMR carrier dissolved in the co-solvent of DW:MeOH (1:2, v/v) was reacted with Cy7-NHS ester under darkness for 24 h. The resulting solution was purified by dialysis in the same manner described above and freeze-dried to obtain MMR-Cy. The targeted imaging agents have been exploited to identify high-risk plaques *in vivo* 23, 28. Next, a theranostic nanodrug, MMR-Lobe-Cy, was fabricated as follows: MMR-Cy and lobeglitazone were completely solubilized in 200 mL of the co-solvent of DW:MeOH (1:3, v/v) for 3 h, dialyzed against DW using a dialysis membrane (MWCO: 6-8 kDa) for 2 days, and lyophilized to yield MMR-Lobe-Cy. The amount of Cy7 in MMR-Cy or MMR-Lobe-Cy was analyzed by measuring the absorbance at 760 nm after MMR-Lobe-Cy (1 mg) or MMR-Cy (1 mg) was clearly dissolved in DMSO (1 mL), respectively. The drug loading content of lobeglitazone in MMR-Lobe-Cy was determined by high-performance liquid chromatography (HPLC) analysis. For particle size analysis, MMR-Lobe-Cy was dispersed in DW using probe-type sonicator and its particle sizes were then analyzed using a Zetasizer 3000 instrument (Malvern Instruments, Malvern, UK). The morphology of MMR-Lobe-Cy was observed using an energy filtering transmission electron microscope (EF-TEM; LEO 912AB OMEGA, Carl Zeiss, Oberkochem, Germany). *In vitro* drug release study was performed using MMR-Lobe-Cy in dialysis membrane (MWCO: 6-9 kDa) immersed in fresh PBS (pH 7.4), and the amount of lobeglitazone released from MMR-Lobe-Cy was measured by HPLC at predetermined time points. The stability of MMR-Lobe-Cy was determined by measuring the hydrodynamic diameters of MMR-Lobe-Cy in PBS and 10% heat-inactivated fetal bovine serum (FBS)-containing Dulbecco's Modified Eagle Medium (DMEM) for 6 days. The detailed experimental methods for the synthesis and characterizations are provided in the supplementary information.

### Preparation of macrophage foam cells and quantification of CD206 expression

Macrophages (RAW264.7 cell; Korean Cell Line Bank, Seoul, South Korea) were incubated in RPMI 1640 medium (Welgene, Gyeongsan, South Korea) supplemented with10% FBS, 100 U/mL penicillin, and 100 μg/mL streptomycin at 37 °C in a humidified 5% CO_2_ atmosphere. Foam cells were established by treating macrophages with LPS (200 ng/mL) and LDL (100 μg/mL) for 24 h 22.

The CD206 mRNA expression in non-stimulated macrophages and foam cells was measured using quantitative PCR (qPCR) assay. Primers that were used are listed in Table S1. Non stimulated RAW264.7 cells and macrophage-derived foam cells were immunostained using primary antibody against CD206 (MR5D3; 1:20; Bio-Rad Laboratories, CA, USA) and secondary antibody conjugated to Alexa Fluor 488 antibody (#405418; 1:50; BioLegend, CA, USA), and then observed under a confocal fluorescence microscope (LSM 900, Carl Zeiss, Oberkochem, Germany). For flow cytometry, samples were incubated with anti-CD206 antibody (MR5D3; 1:10) and Anti-Rat IgG secondary antibody (#405418) according to the manufacturer's instructions, and CD206 expression was analyzed using an LSRFortessa X-20 cell analyzer (BD Bioscience, San Diego, CA, USA). The detailed experimental methods for the qPCR, immunofluorescence and flow cytometry are provided in the supplementary information.

### *In vitro* cellular uptake of MMR-Lobe-Cy to foam cells

Foam cells were treated with MMR-Lobe-Cy (equivalent of 50 μM lobeglitazone) for 1 h. For blocking experiment, foam cells were pre-treated with free mannan (1 mg/mL; a mannose receptor blocker) for 1 h, and additionally treated with MMR-Lobe-Cy (equivalent of 50 μM lobeglitazone) for 1 h. Afterwards, the cells were washed twice with phosphate-buffered saline (PBS). After fixing cells with 3.7% paraformaldehyde, the intracellular internalization was imaged using our custom-built confocal laser-scanning fluorescence microscope (CLSFM) 22. The mean fluorescence intensity of minimum 10 cells was quantified using the ImageJ software (n = 5).

### Anti-inflammatory effects of MMR-Lobe-Cy on foam cells

To verify anti-inflammatory effects in macrophage-derived foam cells, macrophages were pre-treated with MMR-Lobe-Cy (equivalent of 50 μM lobeglitazone) for 2 h, and the cells were additionally treated with LPS (200 ng/mL) and LDL (100 μg/mL) for 24 h. For blocking experiments, mannan (1 mg/mL) was pre-treated 1 h before MMR-Lobe-Cy treatment. Non-stimulated macrophages were used as control cells and macrophage-derived foam cells without MMR-Lobe-Cy treatment as a positive control. After collecting the supernatants from each group, the levels of inflammatory factors including inflammatory chemokine monocyte chemoattractant protein-1 (MCP-1), cytokines IL-1β, and interleukin-6 (IL-6) were determined using a commercial enzyme-linked immunosorbent assay (ELISA) kit (R&D systems, Minneapolis, MN, USA). Cell lysates from each group were tested for enzyme matrix metalloproteinase-9 (MMP-9).

### Tissue distribution and blood half-life of MMR-Lobe-Cy

All animal experiments were approved by the Institutional Animal Care and Use Committee (IACUC) of Korea University College of Medicine (KOREA-2018-0066) and performed in accordance with the National Institutes of Health guide for the care and use of Laboratory animals (NIH Publications No. 8023, revised 1978). For tissue distribution study, MMR-Lobe-Cy (10 mg/kg) was intravenously administered into New Zealand white rabbits (male, 3-month-old; Doo-Yeol Biotech, Seoul, South Korea) (n = 6). At 24 h post-injection, rabbits were sacrificed and the tissues including the liver, spleen, kidney, and lung were excised. Then, the fluorescence signals of the tissues were measured using a fluorescence reflectance imaging (FRI) equipment (excitation: 760 ± 20 nm; Davinch-K Co., Seoul, South Korea) equipped with Cy7 NIRF channel. Also, to determine the blood half-life, a rabbit was also intravenously received 10 mg/kg of MMR-Lobe-Cy. At designated time points (1, 6, 12, 36, 120, and 168 h), 1 mL of blood was collected in BD Vacutainer Heparin Tubes and chilled until analysis. The fluorescence intensity of MMR-Lobe-Cy was measured with the FRI equipment. The blood half-life curve of MMR-Lobe-Cy was fitted using two exponential functions (Prism 7.0; GraphPad, San Diego, CA, USA).

### Atheromatous rabbit models and drug treatments

Atherosclerotic plaques in coronary-sized vessels of rabbits were induced by balloon injury and high cholesterol diet-feeding. After at least 9 to 11 weeks following the balloon injury, total 15 atheromatous rabbits were randomly grouped into MMR-Lobe-Cy (n = 5), oral lobeglitazone (n = 5), and saline control group (n = 5). Rabbits in each group were treated with MMR-Lobe-Cy by intravenous injection (10 mg/kg), lobeglitazone by oral gavage (2 mg/kg), or placebo (saline injection), respectively. Considering the drug loading content and oral bioavailability of lobeglitazone (> 95%) 29, the amount of lobeglitazone in a single dose of MMR-Lobe-Cy is equivalent to that in oral lobeglitazone *per se*. To perform intravascular OCT-NIRF imaging for oral lobeglitazone and control groups, MMR-Cy was additionally treated at a dose of 4 mg/kg *via* intravenous injection so that the NIRF intensity levels in these groups were almost identical to that in the MMR-Lobe-Cy group at baseline. Our previous study reported that the minimum dose of MMR-Cy needed for *in vivo* OCT-NIRF imaging of plaque macrophages was at least 2.5 mg/kg for atheromatous rabbit models 23. FRI analysis demonstrated that fluorescence intensity in 4 mg/kg of MMR-Cy was equivalent to that in 10 mg/kg of MMR-Lobe-Cy. After one week, MMR-Lobe-Cy, lobeglitazone* per se*, saline, and/or MMR-Cy were treated again in the same manner mentioned above. The detailed experimental procedures regarding the development of atheromatous rabbit models were described in the supplementary information.

### Levels of lipids and glucose in blood and body weight

Prior to intravascular imaging, blood samples were collected from the iliac artery and stored in serum separator tube (SST) tubes. Then, the collected blood samples were centrifuged in 4 °C at 3,000 rpm for 5 min to obtain the serum. The levels of total cholesterol (TCHO), triglycerides (TG), LDL, and high-density lipoprotein (HDL) in serum were analyzed using a dry chemistry analyzer (DRI-CHEM NX500, Fuji, Tokyo, Japan) according to the manufacturer's instruction. Blood glucose levels were determined using Accu-Chek glucometer (Roche, Basel, Switzerland). Body weights of the rabbits in all groups were also measured.

### *In vivo* intravascular OCT-NIRF imaging in coronary-sized vessels of atheromatous rabbit models

*In vivo* intravascular OCT-NIRF imaging of plaque lesions was performed as following procedures. At 24 h post-drug treatment, serial imaging was conducted to monitor the NIRF inflammatory signal at baseline (designated as Day 0) and at follow-up time (designated as Day 7). The detailed *in vivo* imaging experiments were mentioned in the supplementary information.

### Analysis of *in vivo* OCT-NIRF imaging

After the raw NIRF data acquisition, the distance-dependent decay of NIRF intensity was computationally compensated based on automated lumen contour segmentation and a pre-determined compensation curve (Figure S3) 30, 31. To eliminate differences in NIRF intensities across rabbits from further analyses, the compensated NIRF signals were normalized as the plaque target-to-background ratio (pTBR) by dividing them by the background value. The background value was determined for each rabbit and calculated by averaging the five lowest per-frame maximum NIRF signals obtained from neighboring normal-looking segments, as previously described 24. We calculated the mean pTBR, a representative inflammatory index of each data, by obtaining the maximum pTBR value for each cross-section and then averaging the values of all cross-sections within a plaque segment. For the detailed intra-animal frame-to-frame NIRF comparison (Day 0 vs. Day 7), the aforementioned maximum pTBR value for each cross-sectional frame was utilized and termed as pTBR/Frame. Corresponding frame pairs between Day 0 and Day 7 were matched by two independent OCT experts, who were blinded to NIRF data, based on side branch orifice, plaque morphology, and plaque size as a morphological guide. Matched frames were randomly sampled at regular intervals from the paired sets of OCT-NIRF images, and 30 pairs selected from each animal were analyzed. For comparison of NIRF intensity changes after treatment among the groups, Δ mean pTBR was calculated by subtracting the mean pTBR at Day 0 from that at Day 7 for each rabbit. To analyze the degree of inflammation reduction following MMR-Lobe-Cy treatment according to baseline inflammation, a total of 150 frame pairs were collected from the MMR-Lobe-Cy group (30 frame pairs in a rabbit and 5 rabbits per group) and analyzed by comparing the relationship between pTBR/Frame values at Day 0 (baseline) and the pTBR/Frame reduction at Day 7 (Δ pTBR/Frame; pTBR/Frame at Day 7 - pTBR/Frame at Day 0). The collected data set was also stratified into three groups according to tertiles of the baseline pTBR/Frame values, and then compared again.

### *Ex vivo* fluorescence reflectance imaging

After acquiring *in vivo* intravascular images, arterial tissues were flushed with saline following sacrifice of rabbits under CO_2_ inhalation. Then, infrarenal aorta was carefully removed while preserving arterial bifurcations. *Ex vivo* NIRF images and signals of the arterial tissues were examined using the FRI system (excitation: 760 ± 20 nm, emission: 832 ± 18 nm). Also, brightfield images were obtained to visualize the plaque formation, and then regions of interests (ROIs) were manually drawn within plaque segments and adjacent normal artery using ImageJ software (National Institute of Health, Bethesda, MD, USA). Likewise, the *ex vivo* mean pTBR was represented as the ratio of the mean NIRF intensity of atheroma and that of normal segment. *In vivo* NIRF 2-dimensional (2D) mappings and *ex vivo* FRI images were matched using the arterial bifurcation, and then the intra-animal correlation between *in vivo* and *ex vivo* NIRF signals was examined along the catheter-axis and aorta-axis, respectively. Both *in vivo* and *ex vivo* NIRF signals from each image were sampled at 0.5 mm interval.

### Histological evaluation of acute anti-inflammatory effects of MMR-Lobe-Cy

After performing the FRI imaging, the arteries were cut transversely at 3 mm intervals, and each tissue segment was frozen in optimal cutting temperature compound (Tissue-Tek®, Sakura Finetek, Tokyo, Japan) at -70 °C. The frozen tissues were cut at 30 μm thickness for fluorescence microscope imaging, and additionally sectioned at 10 μm thickness for further histological validation. Fluorescence microscope (FM) images were acquired using our custom-built CLSFM with an excitation wavelength of 780 nm for Cy7 and 488 nm for auto-fluorescence imaging 22. To observe the overall morphology of the plaques and lipid accumulation, the sectioned samples were stained with hematoxyline and eosin (H&E; Scytek, Logan, UT, USA) and oil-red O (ORO; Scytek), respectively. For immunohistochemistry (IHC) to visualize plaque macrophages, tissue sections were treated with RAM11 anti-macrophage antibody (1:1000 dilution; Dako, Glostrup, Denmark). Moreover, to validate the mechanism for *in vivo* anti-inflammatory effects of MMR-Lobe-Cy, the sections were stained using the following primary antibodies such as MCP-1 (1:500 dilution; Proteintech, Rosemont, IL, USA), ATP-binding cassette subfamily A member 1 (ABCA1, 1:400 dilution; Novus Biologicals, Centennial, CO, USA), and Toll-like receptor-4 (TLR4, 1:200 dilution; Novus Biologicals). RAM11, MCP-1, ABAC1, and TLR4-stained tissues were detected with the Polink-2 HRP Plus Mouse DAB Detection System (GBI Labs, Bothell, WA, USA). After dehydration, clearing, and mounting process, all stained specimens were observed with a light microscope (BX51, Olympus, Tokyo, Japan), and images were acquired with an automated slide scanner (Axio Scan Z1, Zeiss, Oberkochen, Germany).

### *In vivo* evaluation of macrophage contents, protease expression, and collagen contents in plaques after MMR-Lobe-Cy treatment

To further investigate whether MMR-Lobe-Cy treatment affects the changes of macrophages contents, protease expression, and collagen contents in plaques, atheromatous rabbits received with MMR-Lobe-Cy (10 mg/kg, n = 3) or saline (n = 3) once a week for 2 weeks (Figure S5). At Day 14, the harvested aorta tissues were frozen in optimal cutting temperature compound and then sectioned at 10 μm thickness. To examine macrophages and protease expression, the specimens were stained with RAM11 anti-macrophage (1:1000 dilution) and 4A3 anti-MMP-9 (1:100 dilution; Novus Biologicals). Sections were then labeled with the Polink-2 HRP Plus Mouse DAB Detection System and stained tissues were then observed using the light microscope. For collagen contents analysis, the tissue specimens were stained using Picro-Sirius Red (PSR) Stain Kit (SRC-1-IFU; Scytek). Detailed staining method of PSR was described in the supplementary information. PSR-stained tissues were imaged twice with brightfield and polarized microscopy (BX51, Olympus) to identify overall collagen contents and collagen type I, respectively.

### Quantitative analysis of stained tissue images

For quantitative analysis of ORO and PSR-stained sections, we introduced algorithms for the segmentation of the overall and positive-stained tissue section areas. Detailed methods for quantifying ORO and PSR stained images were described in the supplementary information. Also, an open-source digital IHC image analysis software was used to separate the positive-stained areas from IHC images including RAM11, MCP-1, ABCA1, TLR4, and MMP-9 32. This tool can analyze antibody staining intensity from the immunostained images, provide quantitative scale from zero to one, and differentiate the antibody- and counter-stained areas. After segmentation of tissue section area, the pixels with an antibody staining intensity greater than 0.1 in the tissue section area were selected as positive-stained pixels. All selected intensity values were summed to calculate the positive-stained ratio of the immunostained images. The histopathology and IHC quantitation processes were implemented using ImageJ and MATLAB (R2017a; The MathWorks, Natick, MA, USA).

### Western blotting

To explore whether MMR-Lobe-Cy affects the activation of PPARγ and thereby inhibiting TLR4-dependent NF-κB signal pathways, the cultured macrophages (2 × 10^6^ cells per 100 mm dish) were pre-treated with PBS or T0070907 (a potent and selective PPARγ inhibitor; Sigma Aldrich) for 2 h, and they were then treated with MMR-Lobe-Cy (equivalent of 50 μM lobeglitazone) for 1 h. Afterwards, the cells were further exposed with LPS (200 ng/mL) and LDL (100 μg/mL). After 24 h, the cells were lysed with M-PER Mammalian Protein Extraction Reagent (Thermo Scientific, Waltham, MA, USA). After collecting cell lysates by centrifugation at 14,000 rpm for 30 min at 4 °C, cell lysates (20 μg/lane) were separated by 10% sodium dodecyl sulfate (SDS)-polyacrylamide gels, and the proteins were transferred on polyvinylidene difluoride (PVDF) membranes. The membranes were then incubated with the primary antibodies such as PPARγ (NBP1-04676; 1:1000; Novus Biologicals), TLR4 (NBP100-56566; 1:500; Novus Biologicals), total NF-κB (#8242S; 1:1000; Cell Signaling, Danvers, MA, USA), and phosphorylated NF-κB (pNF-κB; #3033S; 1:1000; Cell Signaling) at 4 °C overnight. They were additionally incubated with secondary antibody (1:3000; Santa Cruz Biotechnology Inc., Dallas, TX, USA) for 1 h and then detected using the chemiluminescence western blotting detection kit (Bio-D, Gwangmyeong, South Korea). Quantification of western blot was performed using the ImageJ software by normalizing to loading controls as indicated, and presented as fold changes over the untreated controls.

### Biosafety of MMR-Lobe-Cy

*In vitro* cytotoxicity of MMR-Lobe-Cy in RAW264.7 cells and foam cells were evaluated using cell counting kit-8 (CCK-8, Dojindo Laboratories, Japan) after treatment of MMR-Lobe-Cy containing 0, 1, 5, 10, 20, 50 μM of lobeglitazone for 24 h, and the optical density values were read using a microplate reader at a wavelength of 450 nm. Complete blood count and biochemistry were analyzed in rabbits injected with either saline or a single dose of MMR-Lobe-Cy (10 mg/kg) at 1 day and 1 week after the injection. Red blood cells, hemoglobin, platelets, and white blood cells were analyzed using an automated hematology system (XN-9100; Sysmex Corporation, Kobe, Japan). Aspartate transaminase (AST), alanine transaminase (ALT), blood urea nitrogen (BUN), and creatinine (CRE) were measured on an automatic analyzer (DRI-CHEM NX500i; FUJIFLIM, Japan). The rabbits used for the comprehensive blood testing were sacrificed after 1 week, and their spleen, kidney, liver, and lung were immediately extracted, followed by formalin-fixation, paraffin-sectioning, and H&E staining. The detailed experimental methods for the cytotoxicity, complete blood counting, and biochemistry were provided in the supplementary information.

### Statistical analysis

Statistical analysis was performed with SPSS (Version 23, SPSS Inc., Chicago, IL, USA) and Prism (version 7.0, GraphPad). Quantitative data were expressed as mean ± standard error of the mean. Mean values between three groups were compared *via* a Kruskal-Wallis test followed by the Mann-Whitney U test with Bonferroni correction for post hoc multiple comparisons. Between two groups, a Mann-Wehitney U test was used. The Wilcoxon matched-pairs signed rank test was used to assess the differences in mean pTBR values between at Day 0 and Day 7 from the same rabbit atheroma. Intra-animal frame-to-frame NIRF comparison was analyzed with paired t-test. Relationships between two measurements were assessed by Spearman's or Pearson's correlation analysis according to the Shapiro-Wilk normality test. The *P* values of less than 0.05 were considered statistically significant and all tests were two-tailed.

## Results and Discussion

### Synthesis and characterizations of MMR-Lobe-Cy

**Figure 1:**
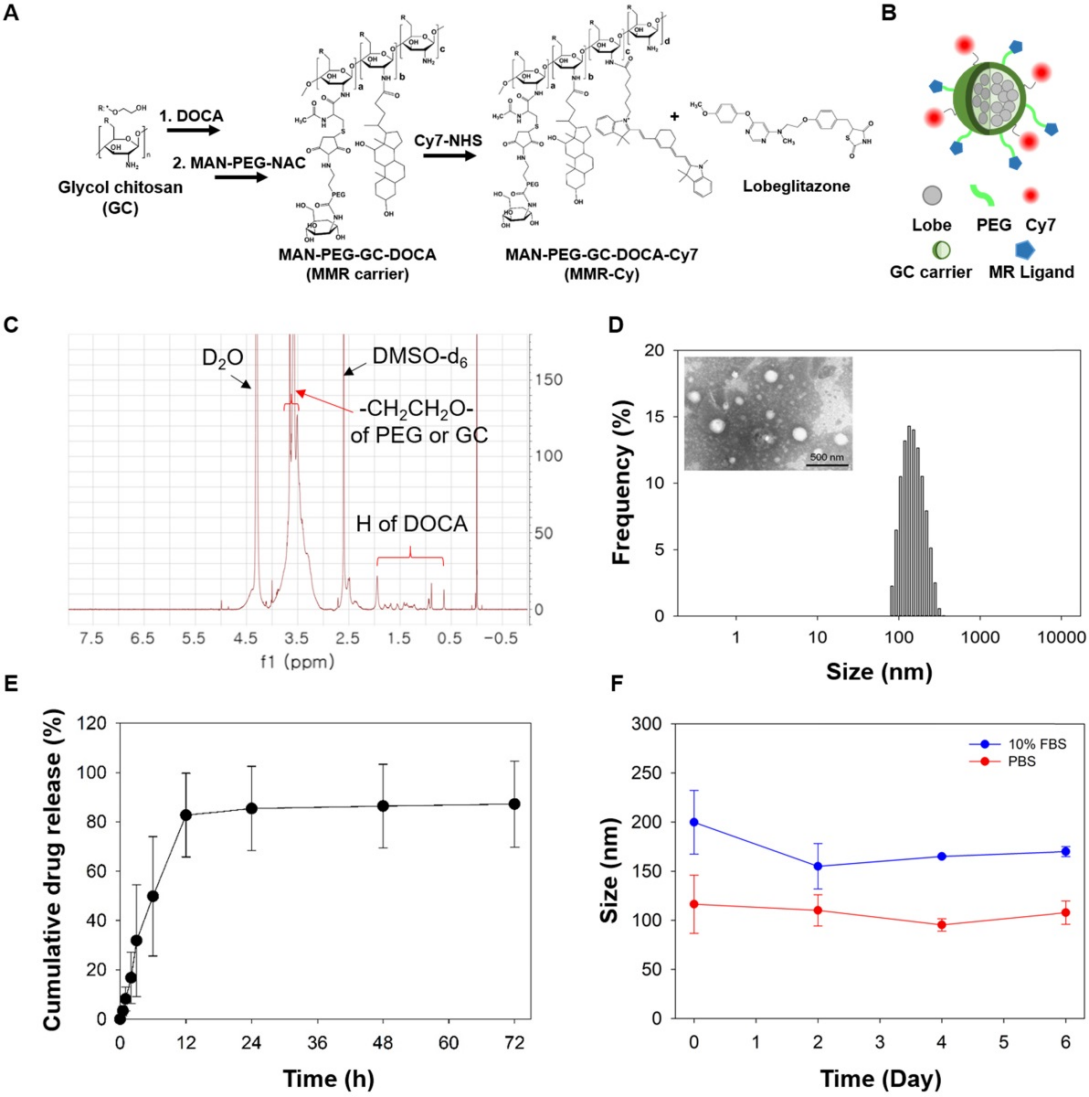
** Characteristics of MMR-Lobe Cy. (A)** Sequential synthetic processes of a theranostic nanodrug, MMR-Lobe-Cy, with strong binding affinity to mannose receptors on macrophages and/or foam cells. MMR-Lobe-Cy was designed to simultaneously detect inflammatory activity and deliver lobeglitazone as an anti-atherogenic drug into high-risk plaque lesions. **(B)** Schematic illustration of MMR-Lobe-Cy. Hydrophobic lobeglitazone molecules were encapsulated within the hydrophobic inner cores of MMR-Cy. **(C)** ^1^H-NMR spectrum of MMR carrier. **(D)** Particle size distribution of MMR-Lobe-Cy dispersed in aqueous solution. Inset: TEM image of MMR-Lobe-Cy. Scale bar, 500 nm. **(E)** *In vitro* release profile of lobeglitazone from MMR-Lobe-Cy. **(F)** The hydrodynamic diameter variation of MMR-Lobe-Cy in PBS (pH 7.4) and 10% FBS-containing DMEM medium.

As an MMR-targeted theranostic agent, the NIRF-emitting MMR carrier was prepared to specifically deliver lobeglitazone into plaque macrophages and scaled up for the translational application to the plaques in coronary-sized arteries. Previous studies have reported that D-mannose had higher binding affinity towards mannose receptors than other monosugars (i.e., N-acetylglucosamine, glucose, xylose, and galactose) 33-37. In this study, we used mannosamine as a specific ligand to mannose receptors because it has the equatorial hydroxyl groups at both the C3 and C4 positions that are the principal determinants of recognition towards mannose receptors (Figure S1). After conjugating it with MAL-PEG-NHS, and as a result, the formed N-acetylmannosamine moiety in MMR-Lobe-Cy had similar structure with mannose (Figure S1D), indicative of specific binding affinity of MMR-Lobe-Cy to MMR. For gram-scale synthesis of MMR-Cy, GC was sequentially reacted with DOCA as a hydrophobic moiety, MAN-PEG-NAC as a specific ligand to the mannose receptor, and Cy7-NHS ester as a NIRF agent (Figure 1A). The conjugation of MAN-PEG-NAC and DOCA with GC was confirmed using the ^1^H-NMR spectrometer. As shown in Figure 1C, we detected proton peaks at 0.63-1.82 ppm from DOCA and methylene, and at 3.3-3.65 ppm from GC and PEG. Then, to obtain MMR-Lobe-Cy, the hydrophobic lobeglitazone as a PPARγ agonist was incorporated into the hydrophobic inner cores of MMR-Cy using a dialysis method (Figure 1A-B). MMR-Lobe-Cy (1 mg) and MMR-Cy (1 mg) contained 0.75 μg and 1.6 μg of Cy7, respectively, as measured by an ultraviolet-visible (UV-Vis) spectrophotometer analysis. The fluorescence intensity of Cy7 in 10 mg of MMR-Lobe-Cy was relatively comparable to that in 4 mg of MMR-Cy as quantitated by the FRI imaging. The loading amount of lobeglitazone within 1 mg of MMR-Lobe-Cy was 0.258 mg as determined by HPLC. The prepared MMR-Lobe-Cy formed the self-assembled nanoparticles in aqueous solution with a hydrodynamic diameter of 207.2 ± 34.79 nm because MMR-Lobe-Cy was composed of hydrophilic outer shell of MAN-PEG-GC and hydrophobic inner cores of DOCA and lobeglitazone (Figure 1B and 1D). The TEM image confirmed the formation of self-assembled nanostructure (Figure 1D). The drug release profile showed that MMR-Lobe-Cy provided a rapid release of lobeglitazone within the first 12 h, followed by a sustained release (Figure 1E). The stability of MMR-Lobe-Cy was tested by measuring the size in PBS and 10% FBS over the 6 days period, and there were no significant changes in the average size of MMR-Lobe-Cy (Figure 1F). These findings suggest that the MMR-Lobe-Cy is stable under biological conditions and avoids unwanted aggregation.

### Specific binding affinity and anti-inflammatory effects of MMR-Lobe-Cy

**Figure 2:**
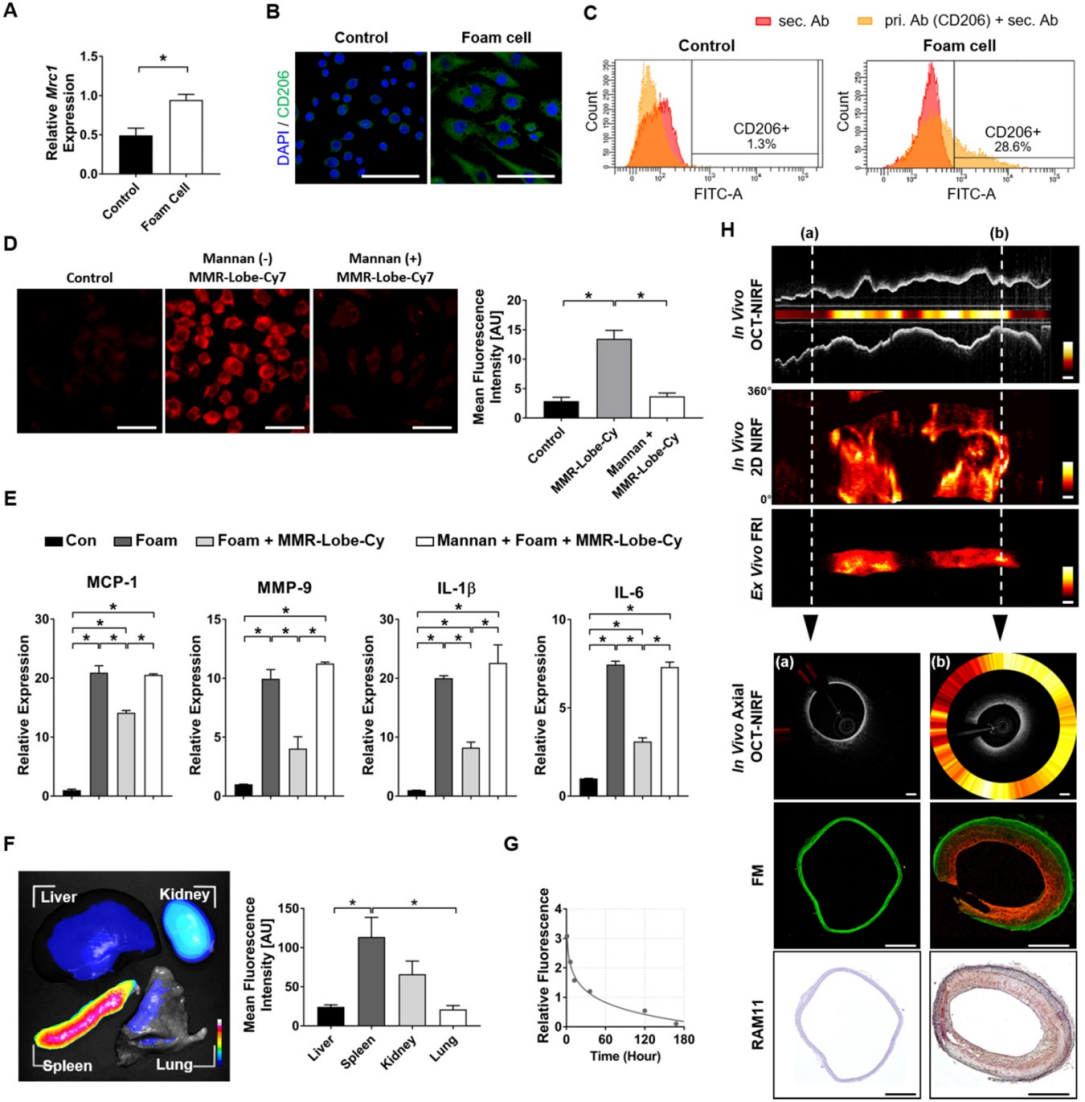
***In vitro* and *in vivo* feasibility study of MMR-Lobe-Cy. (A-C)** Expression of CD206 in foam cells. (A) CD206 (*Mrc1*) mRNA expression in non-stimulated control macrophages and foam cells by qPCR analysis. *P < 0.05 by Mann-Whitney U test. **(B)** Representative immunofluorescence images of non-stimulated control cells (left) and foam cells (right) labeled with CD206 antibody (green). Scale bar, 50 µm. **(C)** Flow cytometry analysis of CD206 expression in control (left) and foam cells (right). Red colored area indicates negative control (staining only with secondary antibody). **(D)** CLSFM images of MMR-Lobe-Cy internalization in foam cells (MMR-derived NIRF in red). The cellular uptake of MMR-Lobe-Cy decreased when mannose receptors on foam cells were blocked with pre-treated free mannan. Scale bars, 30 µm. *P < 0.05 by Kruskal-Wallis test followed by Mann-Whitney U test with Bonferroni correction. **(E)** *In vitro* anti-inflammatory effects of MMR-Lobe-Cy on foam cells as assessed by the expression of inflammatory mediators including MCP-1, MMP-9, IL-1β, and IL-6 by ELISA. Mannose receptor blocking assay with mannan showed the neutralized effects of MMR-lobe-Cy from the foam cells. *P < 0.05 by one-way ANOVA followed by Tukey's post-hoc test. **(F)** Representative *ex vivo* fluorescence imaging of the harvested liver, kidney, spleen, and lung of MMR-Lobe-Cy after 24 h post-injection in rabbits (left), and quantitative fluorescence analysis of tissue biodistribution (n = 6) (right). Data represent the means ± standard error of the mean. *P < 0.05 by Kruskal-Wallis test followed by Mann-Whitney U test with Bonferroni correction. **(G)** Time-dependent blood pharmacokinetics of MMR-Lobe-Cy in a rabbit. After collecting blood sample at predetermined time intervals, NIRF intensity of MMR-Lobe-Cy at each tme step was measured and fitted to calculate the estimated blood half-life time. **(H)** Representative result of *in vivo* intravascular longitudinal OCT-NIRF imaging, *in vivo* 2-dimensional (2D) NIRF mapping, and *ex vivo* FRI. The corresponding *in vivo* axial OCT-NIRF cross-sections, FM images (MMR-derived NIRF in red, autofluorescence in green), and RAM11 immunostained tissue images were designated by white dotted lines; (a) non-injured (normal) and (b) balloon-injured (plaque) arterial segments. Scale bar, 500 µm. Equally windowed.

We first performed qPCR, immunofluorescence staining, and flow cytometry analysis to examine the expressed levels of mannose receptor CD206 in foam cells. As measured by qPCR analysis, CD206 mRNA expression was significantly upregulated in foam cells compared to non-stimulated macrophages (Figure 2A). Immunofluorescence microscopy showed that the CD 206 protein expression was higher in foam cells compared to the control cells (Figure 2B). Flow cytometry analysis revealed that the CD206 expression was significantly increased in foam cells (28.6%) compared to the control cells (1.3%) (Figure 2C).

*In vitro* binding affinity of MMR-Lobe-Cy to atherogenic foam cells was examined using the custom-built CLSFM. MMR-Lobe-Cy was localized to cytoplasm of foam cells, and receptor blocking by pre-treatment with a mannose receptor blocker, mannan, reduced the internalization of MMR-Lobe-Cy into foam cells (Figure 2D), demonstrating that MMR-Lobe-Cy was internalized into foam cells *via* mannose receptor-mediated endocytosis. To assess the anti-inflammatory effects of MMR-Lobe-Cy on foam cells, we analyzed the expression of inflammatory chemokine MCP-1, enzyme MMP-9, cytokines IL-1β and IL-6 in foam cells using the ELISA kit. The expression of MCP-1, MMP-9, IL-1β, and IL-6 were significantly decreased in foam cells treated with MMR-Lobe-Cy, while these changes were reversed by the pre-treatment with mannan, indicating that MMR-Lobe-Cy can more specifically target mannose receptors (Figure 2E). Taken together, the specific binding affinity and anti-inflammatory efficacy of MMR-Lobe-Cy synthesized at gram-scale were consistent with those of our previous MMR-Cy and MMR-Lobe prepared at subgram-scale 22, 23. These *in vitro* data indicate that the modified gram-scale synthesis of MMR-Lobe-Cy was reproducible for targetability to mannose receptors, NIRF visualization, and inhibition of inflammatory activity, implying that the targeted theranostic potential of MMR-Lobe-Cy allows to specifically detect atherogenic foam cells and simultaneously reduce inflammatory activity within the targeted cells.

### Feasibility study for the specific targeting of MMR-Lobe-Cy to atheromatous plaques using *in vivo* intravascular OCT-NIRF imaging system

Next, we validated* in vivo* biodistribution, pharmacokinetics, and feasibility to image plaque macrophages in rabbit atherosclerotic model followed by intravenous injection of MMR-Lobe-Cy. As shown in Figure 2F, the MMR-Lobe-Cy deposition was greatest in the spleen, followed by that in the kidney, liver and lung. The highest uptake of MMR-Lobe-Cy in the spleen might have been attributed to the size of nanoparticle, as larger nanoparticles with diameter greater than 200 nm are typically sequestered by the spleen as a result of mechanical filtration. In addition, the MMR-targeted theranostic agent could accumulate in the kidney by targeting mannose receptors expressed on the mesangial cells 31, by exhibiting a high fluorescence intensity in the kidney. Meanwhile, weaker fluorescence signal was observed in the liver, since attachment of PEG polymer chain to the MMR-targeted agent forms a hydration layer on the particle surface by attracting water molecules and further reduces the hepatic Kupffer cells' ability to phagocytose nanoparticles by opsonin-mediated endocytosis 38-40. Lung uptake of MMR-Lobe-Cy was almost negligible because larger nanomaterials with a size greater than 1000 nm preferentially accumulate in the pulmonary capillary beds by forming a microembolism 41. The half-life of MMR-Lobe-Cy in the blood of rabbit was estimated to 17.4 h according to the fitting result (Figure 2G). Based on these data, we decided 24 h as *in vivo* intravascular imaging time after MMR-Lobe-Cy injection. To investigate whether MMR-Lobe-Cy is able to specifically target rabbit atheroma, we performed *in vivo* intravascular OCT-NIRF imaging at 24 h after the intravenous injection of MMR-Lobe-Cy, and then the imaged aortic vessel was validated using *ex vivo* FRI, CLSFM, and immunostaining (Figure 2H). *In vivo* OCT-NIRF and subsequent *ex vivo* FRI images demonstrated that the NIRF signal at balloon-injured atheroma segments was much stronger than that at non-injured segment. Furthermore, FM and immunostaining imaging clearly supported that MMR-Lobe-Cy specifically targets the macrophages in atheroma regions. The RAM11 positive-staining fraction highly correlated with MMR-Lobe-Cy-derived NIRF signals (*r* = 0.88, *P* < 0.001; Figure S2). Taken together, these results suggest that MMR-Lobe-Cy can identify the inflammatory activity in atheroma lesions in the rabbit aorta as well as distinguish between atheromatous plaques and normal-looking artery.

### *In vivo* serial OCT-NIRF imaging for the evaluation of acute anti-inflammatory effects of MMR-Lobe-Cy in rabbit atheroma

**Figure 3:**
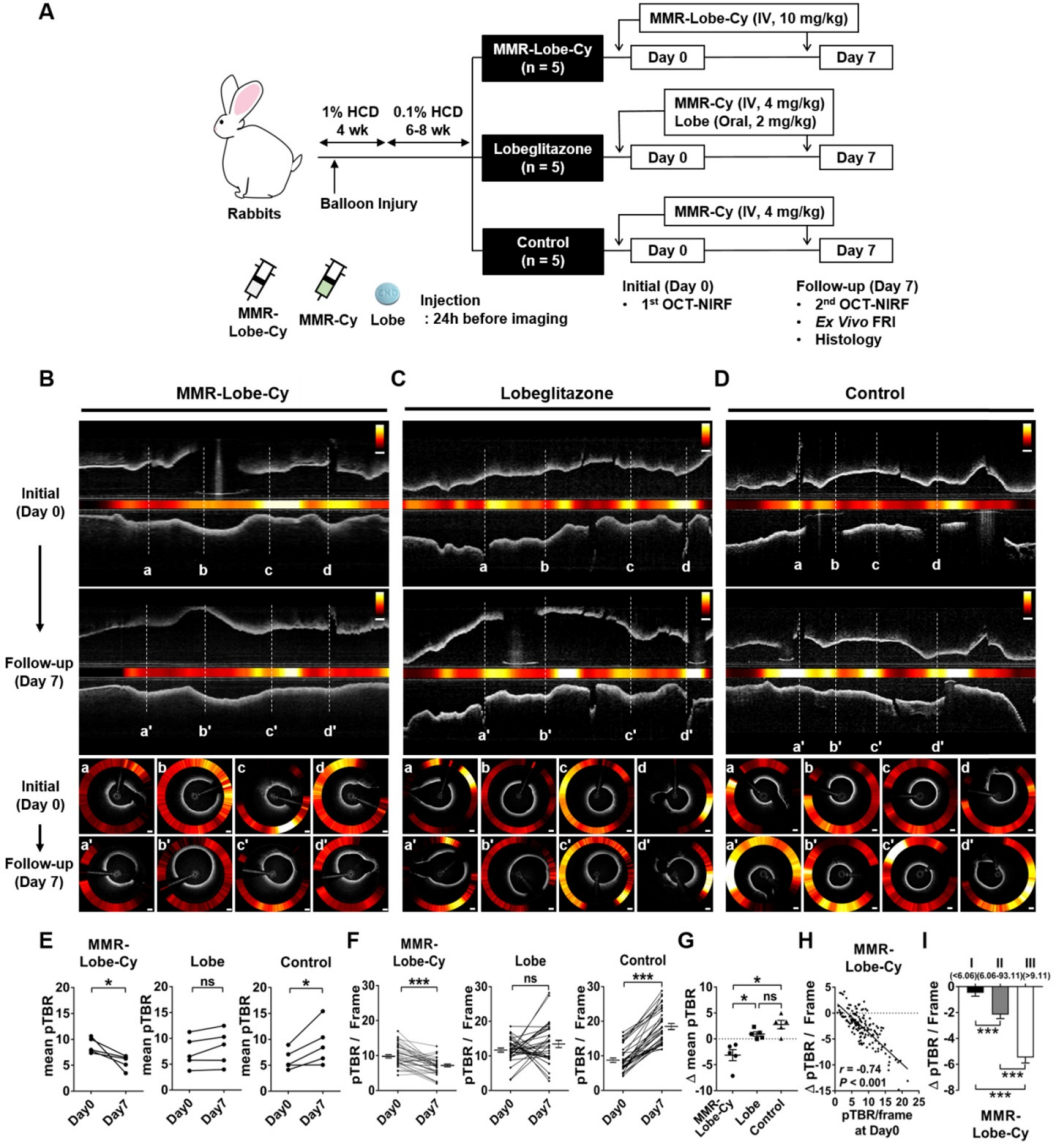
***In vivo* serial OCT-NIRF imaging to evaluate acute inflammation reduction effect of MMR-Lobe-Cy. (A)** Schematic diagrams of the establishment of atheromatous rabbit models (n = 5 per group), drug treatments, and *in vivo* serial OCT-NIRF imaging experiments. Lobe: lobeglitazone. **(B-D)** Representative *in vivo* longitudinal (upper panel) and corresponding axial (lower panel) OCT-NIRF images acquired at initial baseline (Day 0) and follow-up time (Day 7) from groups of (B) MMR-Lobe-Cy, (C) oral lobeglitazone, and (D) saline control. NIRF data were normalized to the plaque target-to-background ratio (pTBR), and then color-coded for the combined visualization. Scale bars, 500 μm. Equally windowed. **(E-I)** Quantitative comparison of the pTBR values among MMR-Lobe-Cy, oral lobeglitazone, and saline control groups. Comparison of (E) mean pTBR and (F) pTBR/Frame from each group, and (G) Δ mean pTBR among three groups. The mean pTBR was obtained by spatially averaging the maximum pTBR value of each frame (pTBR/Frame) within plaque segments. Δ mean pTBR was calculated by subtracting the mean pTBR at Day 0 from that at Day 7. *P < 0.05, **P < 0.01, and ***P < 0.001 by Wilcoxon matched-pairs signed rank test (E), by paired t-test (F), and by Kruskal-Wallis test followed by Mann-Whitney U test with Bonferroni correction (G). ns, non-significant. (H) Spearman's correlation coefficient analysis between initial pTBR/Frame (Day 0) and changes in pTBR/Frame along time (Δ pTBR/Frame; pTBR/Frame at Day 7 - pTBR/Frame at Day 0). (I) The distribution of Δ pTBR/Frame across tertiles of initial pTBR/Frames (Day 0). I, II, and III denote the first (lowest), second, and third (highest) tertile group of the initial pTBR/Frame (n = 30 frames per rabbit, total 150 frames). ***P < 0.001 by Kruskal-Wallis test followed by Mann-Whitney U test with Bonferroni correction.

To assess acute anti-inflammatory effects of MMR-Lobe-Cy *in vivo*, serial OCT-NIRF imaging was performed in rabbit aortic plaque at initial (Day 0) and follow-up time (Day 7) after treatment with MMR-Lobe-Cy, lobeglitazone* per se*, or saline (Figure 3A). In this study, we excluded a MMR c MR carrier had no effects on the inflammation reduction in plaque as well as the activation of the PPARγ pathway 22. In addition, based on the lack of specific binding affinity of non-targeting probe to plaque macrophages 23, the drug delivery efficiency of non-targeting nanodrug was expected to be similar to that of lobeglitazone *per se*. We utilized a dedicated user-interface software that allows real-time display of cross-sectional OCT-NIRF images and further analysis of the corresponding NIRF intensity. The distance-dependent decay of NIRF intensity 31 was computationally compensated based on automated lumen contour segmentation (Figure S3) 30.

In MMR-Lobe-Cy treated rabbits, the mean pTBR, a representative inflammatory index of each data, was significantly reduced at Day 7 compared to Day 0 (Day 0 vs. Day 7: 8.77 ± 0.65 vs. 5.63 ± 0.61, *P* = 0.043; Figure 3B and 3E), whereas, in saline controls, the mean pTBR was even higher at Day 7 compared to baseline (Day 0 vs. Day 7: 6.06 ± 0.87 vs. 9.77 ± 1.82, *P* = 0.043; Figure 3D and 3E). There was no significant difference of mean pTBR between Day 0 and Day 7 in the rabbits treated with lobeglitazone *per se* (Day 0 vs. Day 7: 7.31 ± 1.33 vs. 8.25 ± 1.52, *P* = 0.080; Figure 3C and 3E). Consistently, the changes in plaque inflammation activities were evident in OCT-guided frame matching analyses. MMR-Lobe-Cy treatment significantly reduced pTBR/Frame, the maximum pTBR value for each frame, within the atheroma at Day 7 compared to Day 0 (Day 0 vs. Day 7: 10.03 ± 0.49 vs. 6.76 ± 0.40, *P* < 0.001; a-a' to d-d' in Figure 3B and 3F; Movie S1), while pTBR/Frame increased at Day 7 in the saline-treated rabbit atheroma compared to baseline (Day 0 vs. Day 7: 8.92 ± 0.63 vs. 18.65 ± 0.86, *P* < 0.001; a-a' to d-d' in Figure 3D and 3F; Movie S3). There was no difference of NIRF activities in the matched frame pairs of the oral lobeglitazone group (Day 0 vs. Day 7: 11.71 ± 0.57 vs. 13.47 ± 1.01, *P* = 0.180; a-a' to d-d' in Figure 3C and 3F; Movie S2). The value of Δ mean pTBR, a difference in mean pTBR between Day 0 and Day 7, differed significantly among three treatment groups (MMR-Lobe-Cy vs. oral lobeglitazone vs. control: -3.14 ± 1.07 vs. 0.94 ± 0.41 vs. 2.77 ± 0.81, *P* = 0.005; Figure 3G). The reduction of mean pTBR after treatment was more pronounced in the MMR-Lobe-Cy group compared to oral lobeglitazone or control groups. To further investigate whether the anti-inflammatory efficacy of MMR-Lobe-Cy can be augmented according to the plaque inflammatory levels at baseline, we analyzed the relationship between initial inflammation (pTBR/Frame at Day 0) and inflammation reduction after treatment (Δ pTBR/Frame; Day 7 - Day 0). Intriguingly, the baseline NIRF activity in the plaques negatively correlated with Δ pTBR/Frame, implicating that MMR-Lobe-Cy attenuates plaque inflammation in a macrophage-dependent manner (*r* = -0.74, *P* < 0.001; Figure 3H). As stratified by the baseline inflammation level, a significant difference in Δ pTBR/Frame was noted according to the baseline pTBR/Frame tertiles, indicating that the higher the inflammatory activities at baseline, the greater the reduction of inflammation after MMR-Lobe-Cy treatment (Δ pTBR/Frame: tertile I vs. II vs. III: -0.47 ± 0.26 vs. -2.16 ± 0.31 vs. -5.47 ± 0.42, *P* < 0.001; Figure 3I). In the previous non-targeted PPARγ study, three month administration of pioglitazone significantly suppressed inflammation in atherosclerotic rabbits 42. In contrast, remarkably, our targeted MMR-Lobe-Cy treatment showed a robust decrease in plaque inflammation within 1 week in coronary-sized vessels. Furthermore, acute anti-inflammatory effects of MMR-Lobe-Cy was much more pronounced depending on baseline inflammatory activity. This augmented effect could be explained by targeted property of MMR-Lobe-Cy; localized accumulation of more PPARγ agonist within the macrophage abundant areas of the atheroma. These results indicate that serial *in vivo* assessment using a dual-modal intravascular OCT-NIRF imaging demonstrated a robust reduction of plaque inflammation in coronary-sized arteries within 1 week following MMR-Lobe-Cy administration.

### *Ex vivo* FRI imaging for confirmation of *in vivo* OCT-NIRF findings

**Figure 4:**
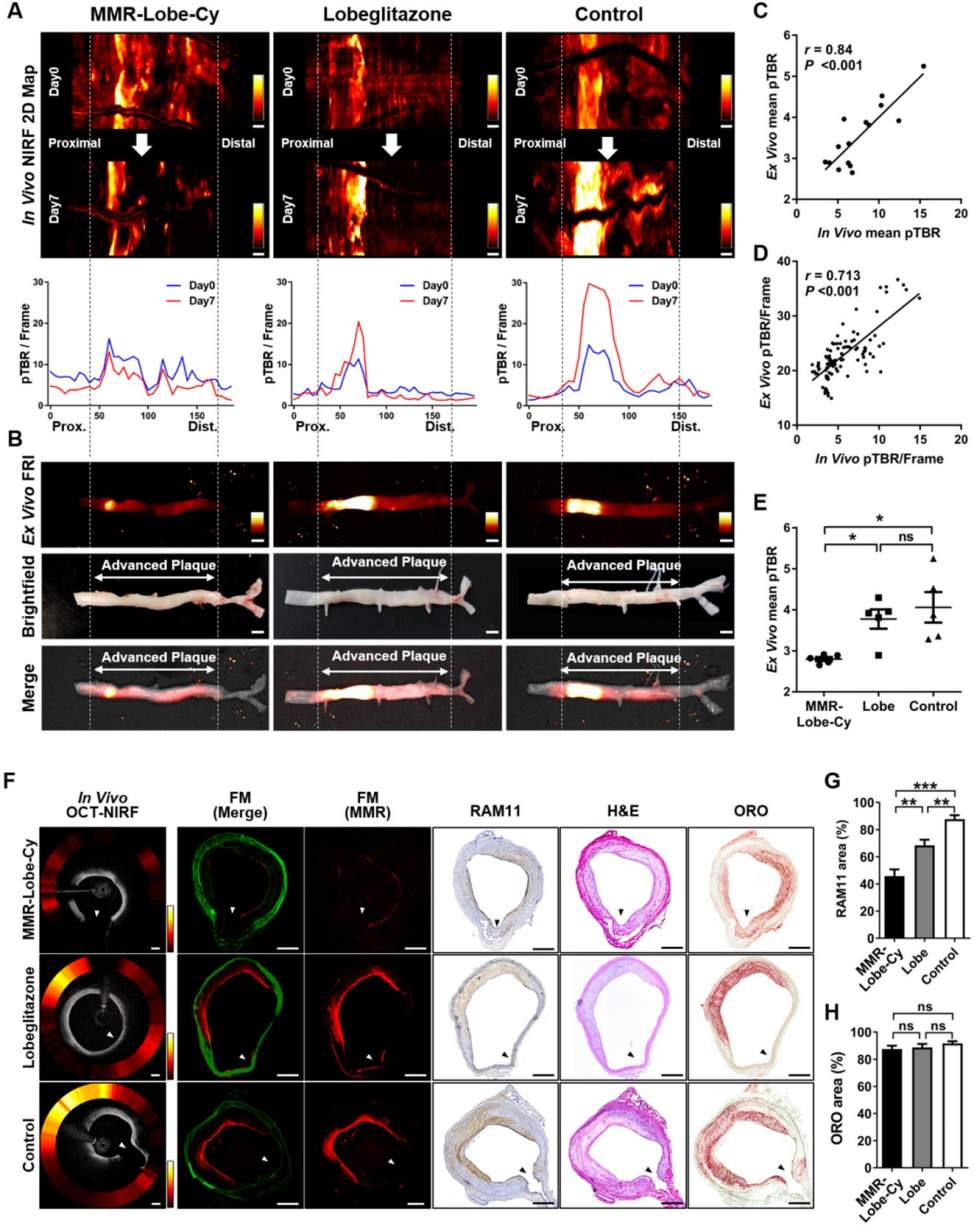
***Ex vivo* FRI NIRF imaging for confirmation of *in vivo* findings. (A)** Comparison of representative *in vivo* NIRF 2-dimensional (2D) mapping between Day 0 (1st row) vs. Day 7 (2nd row), and pTBR/Frame (3rd row) along the pullback direction. NIRF signals decreased after MMR-Lobe-Cy treatment at Day 7 (1st column), whereas the signals tended to increase after administration with saline (3rd column). Prox., proximal; Dist., distal. Scale bars, 4 mm. Equally windowed. **(B)** *Ex vivo* FRI NIRF (top), brightfield (middle), and merged images (bottom) of resected arteries. Scale bars, 4 mm. Equally windowed. **(C)** Pearson correlation analysis of *in vivo* and *ex vivo* mean pTBR. **(D)** A linear relationship between *in vivo* vs. *ex vivo* pTBR/Frame values of the corresponding sites of the artery (*r*: Spearman correlation coefficient). **(E)** Quantitative comparison of *ex vivo* mean pTBR comparison among each group. *P < 0.05 by Kruskal-Wallis test followed by Mann-Whitney U test with Bonferroni correction. ns, non-significant. **(F-H) Co**-relationship between *in vivo* axial OCT-NIRF images and histological validation. (F) The representative *in vivo* axial OCT-NIRF cross-sectional images of each group correspond with the FM images (MMR-derived NIRF in red, autofluorescence in green), macrophage immunostaining (RAM11), haematoxylin and eosin staining (H&E), and oil red O (ORO) lipid staining, respectively. Scale bars, 500 µm. Equally windowed. (G) Quantitative analysis of RAM11-positive macrophage areas of each treatment group including MMR-Lobe-Cy, oral lobeglitazone, and saline control. (H) ORO staining area comparison for lipid composition of each group. **P < 0.01, ***P < 0.001 by Kruskal-Wallis test followed by Mann-Whitney U test with Bonferroni correction. ns, non-significant.

*In vivo* NIRF 2D mapping results were also acquired at initial (Day 0) and follow-up time (Day 7). Unlike the oral lobeglitazone and saline control groups, MMR-Lobe-Cy could effectively reduce the pTBR/Frame values within plaque segments, showing its prominent anti-inflammatory effects (Figure 4A). To further validate these *in vivo* findings, OCT-NIRF imaged aortas were resected, and then *ex vivo* FRI was performed. A strong NIRF signal was detected in the advanced atheroma portion of the brightfield images (Figure 4B). This *ex vivo* NIRF signals well matched with the *in vivo* NIRF 2D mapping at Day 7 (Figure 4A and 4B). The *in vivo* mean pTBR from OCT-NIRF images at Day 7 significantly correlated with *ex vivo* mean pTBR from FRI (*r* = 0.84, *P* < 0.001; Figure 4C). Likewise, the *in vivo* and *ex vivo* pTBR/Frame values along the pullback direction in each artery were well co-localized (*r* = 0.713, *P* < 0.001; Figure 4D). The *ex vivo* mean pTBR in MMR-Lobe-Cy group was significantly lower compared to both oral lobeglitazone and saline control groups as well (MMR-Lobe-Cy vs. oral lobeglitazone vs. control, 2.80 ± 0.05 vs. 3.77 ± 0.24 vs. 4.06 ± 0.37, *P* = 0.005; Figure 4E). Consistent with the *in vivo* findings, this evidence implies that *ex vivo* imaging analysis corroborated the specific targeting of MMR-Lobe-Cy to atheroma and reduction of inflammatory activity within plaques after MMR-Lobe-Cy treatment.

### Correlative fluorescence microscopy and histological validation

*In vivo* axial OCT-NIRF images obviously supported that MMR-Lobe-Cy had prominent acute anti-inflammatory effects on rabbit atheroma compared to oral lobeglitazone or saline control groups (Figure 4F). To further confirm the prominent anti-inflammatory effects of MMR-Lobe-Cy, FM imaging and histological validation were carried out using the resected aortae from each group. The NIRF signals from *in vivo* axial OCT-NIRF images well corresponded to those on FM images from the matched sections (Figure 4F). Consistent with *in vivo* OCT-NIRF imaging, the NIRF signals from FM images in MMR-Lobe-Cy group was much weaker than those in oral lobeglitazone or saline control groups. On the FM images and corresponding immunohistochemistry of RAM11, strong NIRF signals in oral lobeglitazone or saline control groups were detected at the macrophage-abundant areas of the plaques, whereas weak NIRF signal in MMR-Lobe-Cy were observed at macrophage-scarce areas (Figure 4F). The amount of plaque macrophages quantitatively decreased in the MMR-Lobe-Cy group by 33.2% vs. oral lobeligtazone (*P* = 0.006) and by 47.9% vs. control (*P* < 0.001) (Figure 4G). The RAM11 positive area was reduced by 22.0% in the oral lobeglitazone group compared to the saline control group (*P* = 0.003) (Figure 4G). Further histological validation was performed with ORO lipid staining to assess the cholesterol efflux activity by PPARγ 22, however, no significant difference was observed between the groups (*P* = 0.640) (Figure 4H). These results imply that the short-term administration of MMR-Lobe-Cy has prominent anti-inflammatory effects on atheroma, but is insufficient to reduce lipid contents in the plaque within a short period time.

### Inhibition of TLR4/NF-κB by MMR-Lobe-Cy

**Figure 5:**
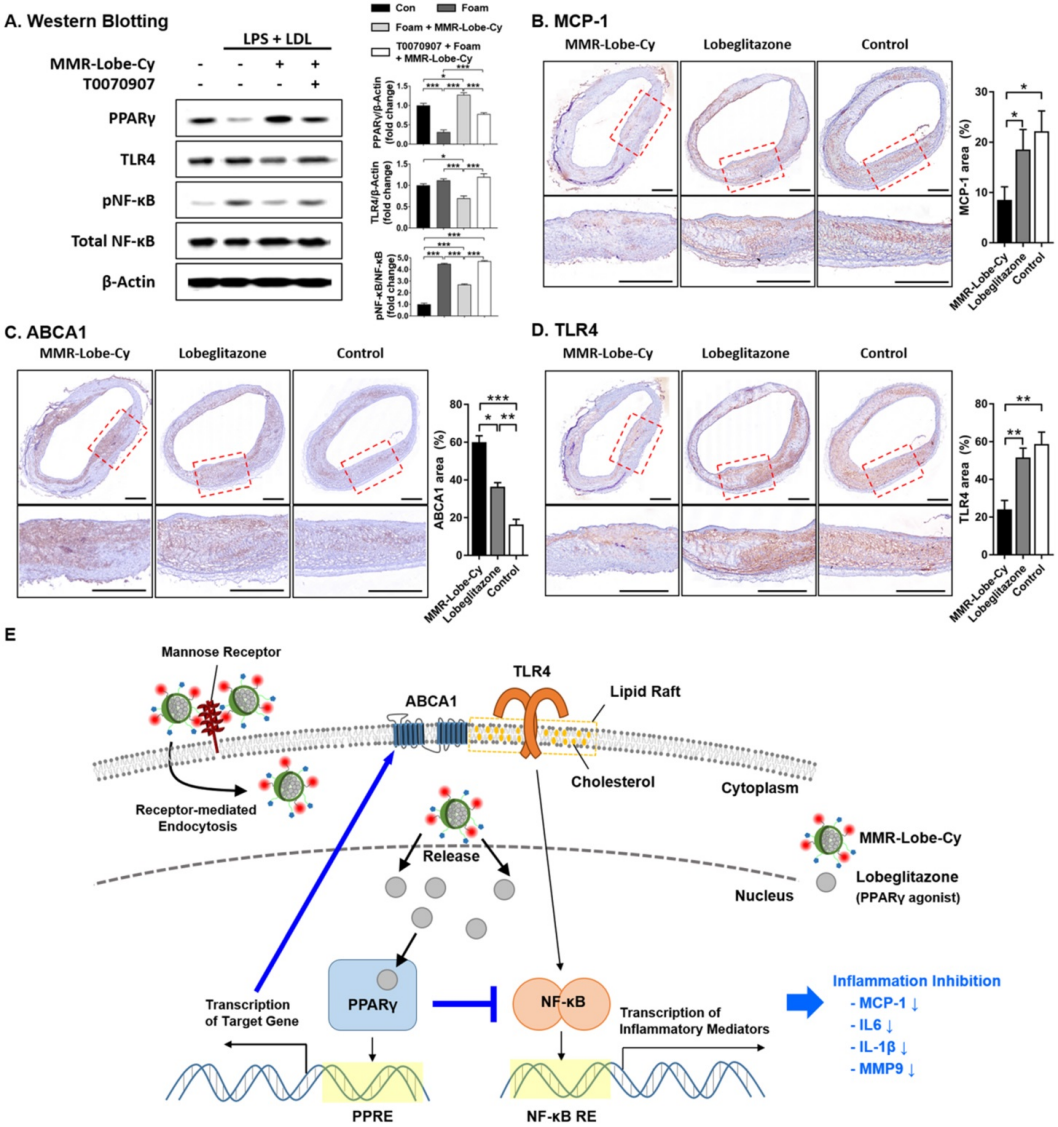
** Inhibition of TLR4/NF-κB signaling pathway by MMR-Lobe-Cy. (A)** Western blot analysis of PPARγ, TLR4, phosphorylated NF-κB (pNF-κB), and total NF-κB. PBS- or T0070907 PPARγ inhibitor pre-treated macrophages were treated with MMR-Lobe-Cy, followed by stimulation with LPS and LDL. TLR4, Toll-like receptor-4; NF-κB, nuclear factor-κB. Quantification of PPARγ, pNF-κB, and TLR4 was normalized to β-Actin, NF-κB, and β-Actin, respectively, and presented as fold changes over the controls. **(B-D)** Comparison of MCP-1, ABCA1, and TLR4 expression in rabbit atheroma between the groups of MMR-Lobe-Cy*,* oral lobeglitazone, and saline control. Representative immunohistochemical images and quantitative comparison of (B) MCP-1, (C) ABCA1, and (D) TLR4 expression in rabbit atheroma after drug treatments. Scale bars, 500 µm. *P <0.05, **P < 0.01, ***P < 0.001 by Kruskal-Wallis test followed by Mann-Whitney U test with Bonferroni correction. **(E)** Model for mechanisms of inflammation inhibition by MMR-Lobe-Cy in plaque macrophages. MMR-Lobe-Cy provides specific delivery of lobeglitazone to plaque macrophages *via* receptor-mediated endocytosis. Released lobeglitazone activates PPARγ, binds to PPAR response element (PPRE) in target genes, and its transcription upregulates ABCA1 that induces cholesterol efflux. In addition, activated PPARγ represses the transcription of NF-κB that binds to DNA through NF-κB response element (NF-κB RE), and then inhibits the production of inflammatory mediators through blocking of TLR4/NF-κB pathway.

Previous studies reported that PPARγ agonists have anti-inflammatory effects by inhibiting the TLR4/NF-κB signaling pathway, the key regulator of inflammatory responses 43-45. To investigate whether the PPARγ activation through the specific delivery of lobeglitazone using MMR-Lobe-Cy negatively regulates the TLR4/NF-κB signaling pathway in atherogenic foam cells, we performed western blotting to assess the protein expression of PPARγ, TLR4, NF-κB and pNF-κB in foam cells treated with MMR-Lobe-Cy. MMR-Lobe-Cy increased PPARγ expression and negatively regulated TLR4 and pNF-κB expressions in foam cells, and these actions were blocked by a PPARγ antagonist, T0070907 (Figure 5A). This finding suggests that MMR-Lobe-Cy inhibits TLR4/NF-κB signaling pathway through PPARγ activation in macrophage foam cells.

Monocyte infiltration mainly contributes to accumulation of plaque macrophages. The expression of MCP-1 is enhanced by macrophages 46 and reduced in atherosclerotic plaques by treatment of PPARγ agonist 47. To further demonstrate *in vivo* effects of MMR-Lobe-Cy on MCP-1 expression, we assessed the expression of MCP-1 in macrophage-rich atheroma treated with MMR-Lobe-Cy. MMR-Lobe-Cy significantly inhibited the expression of MCP-1 compared to both oral lobeglitazone and control groups (MCP-1 area (%) between MMR-Lobe-Cy vs. oral lobeglitazone, *P* = 0.032; MMR-Lobe-Cy vs. control, *P* = 0.017; oral lobeglitazone vs. control, *P* = 1.00) (Figure 5B), suggesting that MMR-Lobe-Cy attenuated inflammation in the plaques partly *via* reduction of MCP-1, which is primarily responsible for monocyte chemotaxis and infiltration in atherosclerosis 48, 49. As ABCA1 expression reorganizes plasma membrane lipid rafts at the site where signaling cascade is concentrated 50, we investigated the effects of MMR-Lobe-Cy on the expression of ABCA1 and TLR4 within the atheroma. MMR-Lobe-Cy significantly increased the expression of ABCA1 (MMR-Lobe-Cy vs. oral lobeglitazone, *P* = 0.028; MMR-Lobe-Cy vs. control, *P* < 0.001; oral lobeglitazone vs. control, *P* = 0.003) (Figure 5C) and suppressed the expression of TLR4 within the plaque (MMR-Lobe-Cy vs. oral lobeglitazone, *P* = 0.005; MMR-Lobe-Cy vs. control, *P* = 0.001; oral lobeglitazone vs. control, *P* = 1.00) (Figure 5D). Notably, MMR-Lobe-Cy treatment in macrophage foam cells elicits the expression of PPARγ and blocked the expression of TLR4 and pNF-κB. This is consistent with the previous reports that PPARγ agonists repress the transcriptional activity of NF-κB 51-53 and regulate inflammation through TLR4 54. Furthermore, MMR-Lobe-Cy treatment in rabbit atheroma significantly enhanced ABCA1 levels and inhibited TLR4 expression compared to oral lobeglitazone and saline control. As PPARγ activation enhanced the ABCA1 expression and attenuated the TLR4 expression through modulation of macrophage membrane lipid rafts that coordinate various signaling pathways 50, 55, 56, MMR-Lobe-Cy taken up by plaque macrophages could specifically activate PPARγ pathway, resulting in acute suppression of inflammatory activity through blocking of NF-κB and ABCA1-mediated inhibition of TLR4/NF-κB signaling. Taken together, these results suggest the underlying mechanism of MMR-Lobe-Cy inducible anti-inflammatory effects on atheroma, supported by increased ABCA1 expression and blocked TLR4/NF-κB-dependent inflammatory pathway, and also reduction of MCP-1 (Figure 5E).

### Metabolic parameters following MMR-Lobe-Cy treatment

Body weight, blood glucose levels, and lipid profiles of the subjects were measured to assess systemic metabolic influences of MMR-Lobe-Cy. There were no significant differences in body weights, blood glucose levels, and lipid levels among the three groups at the end of the experiments (Figure S4), indicating that MMR-Lobe-Cy does not cause metabolic side effects.

### Biosafety of MMR-Lobe-Cy

**Figure 6:**
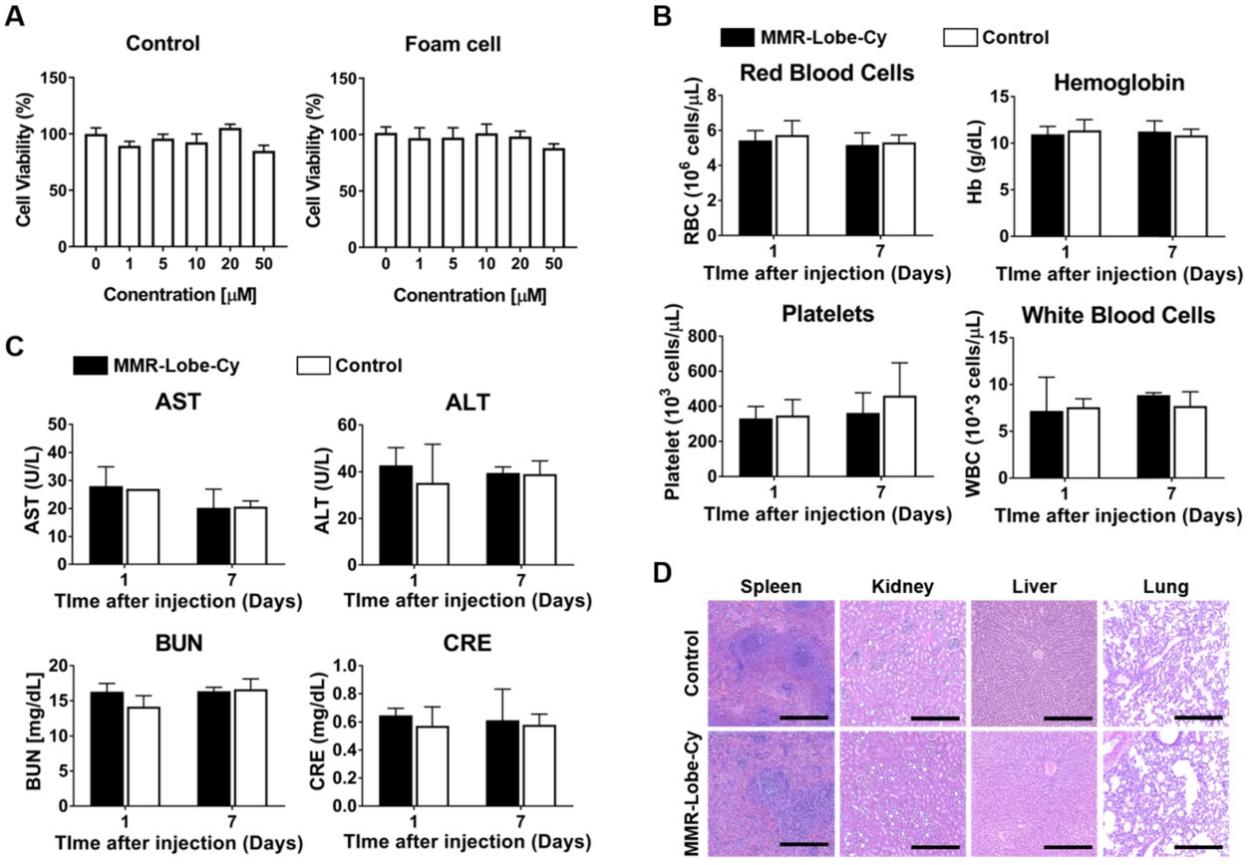
** Biosafety assessment of MMR-Lobe-Cy. (A)** Effect of MMR-Lobe-Cy on the viability of control cells (RAW264.7 without treatment) and foam cells (RAW264.7 with LPS and LDL). The concentration refers to the amount of loaded lobeglitazone. Data are presented as mean ± standard error of the mean (n = 4). **(B, C)** Eight rabbits were injected with either normal saline (n = 4) or 10 mg/kg of MMR-Lobe-Cy (n = 4). Blood was collected at 1 and 7 days after the injection. (B) Complete blood counts showed no effects of MMR-Lobe-Cy on red blood cells, hemoglobin, platelets and white blood cells. (C) Biochemistry analysis of AST, ALT, BUN and CRE. **(D)** Histological analysis of spleen, kidney, liver and lung from rabbits 7 days after the MMR-Lobe-Cy injection. Scale bar, 100 µm. *P*-values were calculated by Mann-Whitney U tests (two sided). RBC, red blood cells; Hb, hemoglobin; WBC, white blood cell; AST, aspartate aminotransferase; ALT, alanine transaminase; BUN, blood urea nitrogen; CRE, creatinine.

To evaluate the biosafety of MMR-Lobe-Cy, we performed cell viability assay, comprehensive blood test, and histological analysis. As shown in Figure 6A, by CCK-8 assay, MMR-Lobe-Cy was not toxic up to a concentration containing 50 μM of lobeglitazone in both macrophages and foam cells (*P* > 0.05). Complete blood count data collected twice from the rabbits injected with either saline or a single dose of MMR-Lobe-Cy (10 mg/kg) at 1 day and 1 week after the injection showed no significant differences in white blood cells, red blood cells, hemoglobin, and platelets between saline vs. MMR-Lobe-Cy treated rabbits (Figure 6B). Blood chemistry analysis also revealed no signs of hepatic or renal toxicity in MMR-Lobe-Cy group as compared to the control group (Figure 6C). The rabbits used for the comprehensive blood testing were sacrificed after 1 week, and their spleen, kidney, liver, and lung were immediately harvested for the histopathological examination. As shown in Figure 6D, there were no evidence of damage or impairment in the tissues. These data indicate that MMR-Lobe-Cy at a dose of 10 mg/kg causes no harmful effects on liver, kidney and other major organs. The backbone of MMR-Lobe-Cy was composed of biologically safe components, including DOCA, PEG, and chitosan. DOCA is approved for submental fat reduction 57, and PEGlyated lipid is currently used as an excipient in two types of mRNA-based SARS-CoV-2 vaccines manufactured by the Moderna and Pfizer-BioNTech 58. Chitosan is considered as a GRAS (Generally Recognized as Safe) compound, and numbers of clinical studies are ongoing to investigate the biocompatibility, safety, and efficacy of chitosan-based nanoparticles 59, 60. Furthermore, our toxicity assay has provided that MMR-Lobe-Cy has negligible effects on body weight, hematological, biochemical, and histological profiles *in vivo* (Figure 6). While further studies are required to clarify the quality, safety, and efficacy of our scale-up agent, we believe that MMR-Lobe-Cy has high translational potential for the clinical application.

### Ultimate shift of plaque composition to a more stable phenotype by MMR-Lobe-Cy

**Figure 7:**
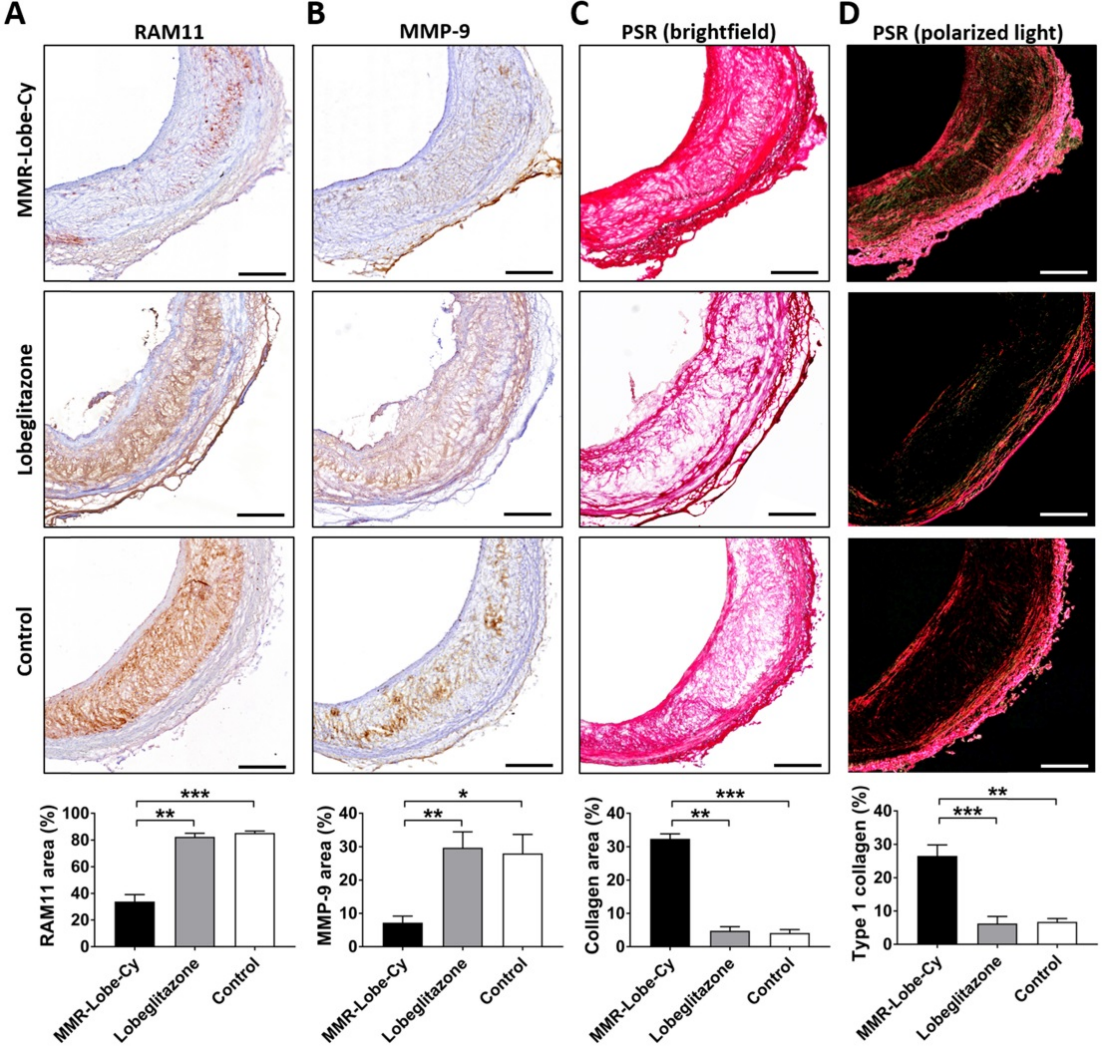
***In vivo* evaluation of macrophage contents, protease expression, and collagen contents in rabbit atheroma after 14 days of MMR-Lobe-Cy treatment.** Comparison of **(A)** RAM11 macrophage contents and **(B)** MMP-9 expression in rabbit atheroma. PSR-stained plaques imaged with **(C)** brightfield microscopy to show total collagen contents and **(D)** polarized microscopy to evaluate collagen type I contents. n = 3 segments per rabbit, n = 3 rabbits per group. Scale bar, 200 µm. *P < 0.05, **P < 0.01, and ***P < 0.001 by Kruskal-Wallis test followed by Mann-Whitney U test with Bonferroni correction.

To evaluate whether the anti-inflammatory effects of MMR-Lobe-Cy influence plaque composition, we performed the immunostainings of RAM11 and MMP-9, and the histological staining of collagen in sister sections of the rabbits treated with MMR-Lobe-Cy, lobeglitazone or saline at Day 14 (Figure S5). The atheroma immunostained with anti-RAM11 antibody exhibited a robust macrophage depletion in the MMR-Lobe-Cy treated rabbits (% area, 33.89 ± 5.24) as compared to oral lobeglitazone (% area, 82.48 ± 2.63, *P* < 0.01) and control groups (% area, 85.39 ± 1.40, *P* < 0.001) (Figure 7A). Similarly, MMP-9 expression decreased markedly within the plaques of MMR-Lobe-Cy group (% area, 7.21 ± 1.96) compared to those of oral lobeglitazone (% area, 29.72 ± 4.78, *P* < 0.01) and control groups (% area, 28.03 ± 5.68, *P* < 0.05) (Figure 7B). Intriguingly, brightfield images of PSR staining showed a greater amount of collagen within the plaques of MMR-Lobe-Cy group (% area, 32.37 ± 1.52) than those of oral lobeglitazone (% area, 4.78 ± 1.22, *P* < 0.01) and control groups (% area, 4.12 ± 1.03, *P* < 0.001) (Figure 7C). These changes of collagen contents were observed only at Day 14, whereas there were no changes in collagen contents at Day 7. In particular, the amount of collagen type I, as assessed by polarized light microscopic analysis, was significantly increased in the MMR-Lobe-Cy-treated plaques (% area, 26.56 ± 3.30) compared to those in the lobeglitazone (% area, 6.24 ± 2.14, *P* < 0.001) and saline-treated lesions (% area, 6.74 ± 1.01, *P* < 0.01) (Figure 7D). It is well-known that infiltrated macrophages in plaques are the key sources of matrix metalloproteinases which contribute to the degradation of interstitial collagen, leading to plaque rupture 12, 61. Interestingly, at 2 weeks of MMR-Lobe-Cy treatment, *in vivo* histological staining data showed a diminished macrophages and MMP-9 expression with an increase of type I collagen matrix, whereas similar effects of oral pioglitazone on murine atheroma required a long-term administration over 8 weeks 62. These findings suggest that the plaques in MMR-Lobe-Cy treated group were more fibrotic compared to those in oral lobegltiazone and controls, and accordingly, it appears that current MMR-Lobe-Cy treatment substantially enhances the plaque stability by reduction of inflammation including macrophage infiltration and matrix metalloproteinase activity, and also increase of collagen contents, particularly, type I. Taken together, we suggest that MMR-Lobe-Cy treatment enabling a rapid shift of high-risk plaque into a more stabilized atheroma could be a promising therapeutic opportunity for cardiovascular disease, particularly in the acute settings.

## Limitations

Despite providing intriguing data, the current study has still limitations. First, MMRs could be associated with alternatively activated macrophages, although they are also present in high-risk plaques such as thin-cap fibroatheroma 63, plaques with neovascularization and intraplaque hemorrhages 64, 65. However, this dichroic classification which chiefly relies on cell surface markers is still an issue due to a plasticity of both surface markers and M1/M2 markers 66. Additionally, a heterogeneous spectrum of macrophage phenotypes between anti- and pro-inflammatory poles has imperfect implications for the translation of mouse to large animals 67, 68. Considering that MMR-Lobe-Cy ultimately transformed the high-risk plaque to a more stable one, MMR-positive macrophages may serve as an effective carrier to deliver the PPARγ agonist to the high-risk plaques beyond M2 macrophages and we speculate that MMR-Lobe-Cy treatment has overall favorable effects on the inflamed coronary atheroma, albeit further research regarding the long-term effects of MMR-Lobe on macrophage plasticity is needed. Second, rabbits were fed with normal diet after treatment of MMR-Lobe-Cy to avoid the cholesterol-mediated hepatotoxicity. Given that the low-density lipoprotein cholesterol (LDL-C) induces inflammation in the arterial wall 69, 70, switching from high cholesterol to normal diet could potentially reduce LDL-C levels and further promote attenuation of plaque inflammation. Despite concerns about normal diet supplementation, average serum LDL-cholesterol levels of these rabbits after 1 week were 376 mg/dL (Figure S4), which was as nearly twice as much as even severe hypercholesterolemic patients (> 190 mg/dL) 71. Furthermore, by serial OCT-NIRF imagings, inflammatory activity along the aortic wall significantly increased in the same rabbit models injected with saline (Figure 3D and 3E). Taken together, we assume that the acute anti-inflammatory effects were mainly attributed to the treatment of MMR-Lobe-Cy rather than a short period dietary change.

## Conclusion

**Figure 8:**
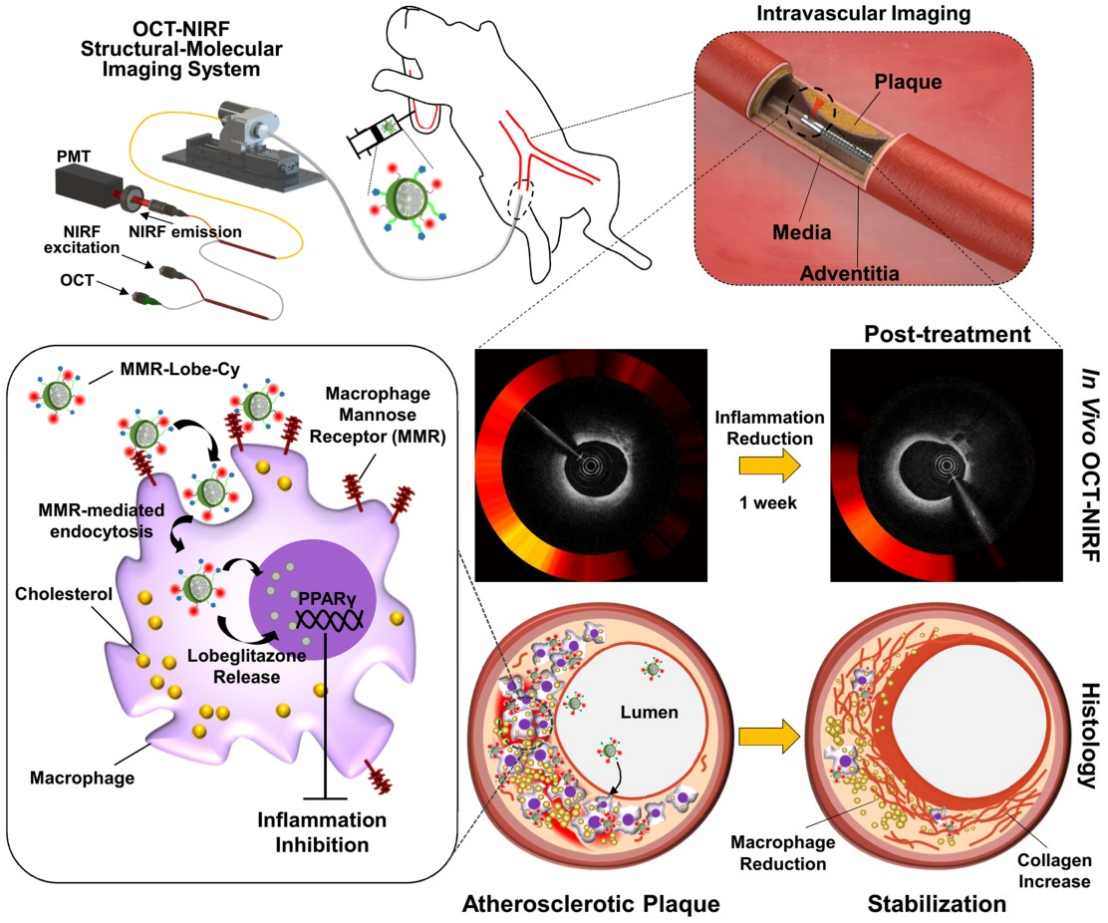
** Schematic illustration of *in vivo* OCT-NIRF imaging for monitoring vascular inflammation and simultaneous drug delivery for plaque stabilization using a theranostic nanodrug,** MMR-Lobe-Cy. *In vivo* intravascular OCT-NIRF catheter imaging was performed to monitor early inflammation responses in plaques in coronary-sized arteries after MMR-Lobe-Cy treatment in atheromatous rabbit. Due to the strong binding affinity to mannose receptors, MMR-Lobe-Cy is internalized into plaque macrophages and foam cells. After the uptake into the cells, MMR-Lobe-Cy releases the drug (lobeglitazone) and activates PPARγ pathway, leading to the inflammation inhibition in plaque lesions. Rapid reduction of inflammation by MMR-Lobe-Cy leads to subsequently change the atheroma into a macrophage-poor and collagen-rich stable plaque phenotype. PMT, photomultiplier tube.

In conclusion, our targeted theranostic agent, MMR-Lobe-Cy, showed a robust acute anti-inflammatory effects on inflamed plaque in coronary-sized arteries by inhibition of TLR4/NF-κB pathway, and shifted the plaque composition to a stable phenotype, which was successfully assessed by our integrated OCT-NIRF imaging (Figure 8). This approach could be an innovative strategy for management of cardiovascular disease in acute settings, with translational implications for high-risk plaques.

## Supplementary Material

Supplementary figures and tables.

Supplementary movie S1.

Supplementary movie S2.

Supplementary movie S3.

## Abbreviations

MMR: macrophage mannose receptor
Lobe: lobeglitazone
PPARγ: peroxisome proliferator-activated gamma
OCT: optical coherence tomography
NIRF: near-infrared fluorescence
FRI: fluorescence reflectance imaging
TLR4: toll-like receptor-4
NF-κB: nuclear factor-κB
PET: positron emission tomography
CT: computed tomography
LPS: lipopolysaccharide
LDL: low-density lipoprotein
CLSFM: confocal laser-scanning fluorescence microscope
MCP-1: monocyte chemoattractant protein-1
MMP-9: matrix metalloproteinase-9
IL-1β: interleukin-1β
IL-6: interleukin-6
TCHO: total cholesterol
TG: triglycerides
HDL: high-density lipoprotein
pTBR: plaque target-to-background ratio
FM: fluorescence microscope
IHC: immunohistochemistry
ABCA1: ATP-binding cassette subfamily A member 1
UV-Vis: ultraviolet-visible
PPRE: PPAR response element
NF-κB RE: NF-κB response element
