# Supplementary material for: Macrophage targeted theranostic strategy for accurate detection and rapid stabilization of the inflamed high-risk plaque
Source: Theranostics. 2021 Aug 18;11(18):8874–93. doi: 10.7150/thno.59759 (PMC8419038; doi:10.7150/thno.59759)
Supplement: Supplementary file 1 — Supplementary figures and tables. [file thnov11p8874s1.pdf]

Macrophage targeted theranostic strategy for accurate detection and rapid stabilization of the inflamed high-risk plaque

Joon Woo Song[1,#], Hyeong Soo Nam[2,#], Jae Won Ahn[3,#], Hyun-Sang Park[2], Dong Oh Kang[1], Hyun Jung Kim[1], Yeon Hoon Kim[2], Jeongmoo Han[2], Jah Yeon Choi[1], Seung-Yul Lee[1], Sunwon Kim[1], Wang-Yuhl Oh[2], Hongki Yoo[2,*], Kyeongsoon Park[3,*], Jin Won Kim[1,*]

[1]Multimodal Imaging and Theranostic Lab., Cardiovascular Center, Korea University Guro Hospital, Seoul, South Korea

[2]Department of Mechanical Engineering, Korea Advanced Institute of Science and Technology, Daejeon, South Korea

[3]Department of Systems Biotechnology, Chung-Ang University, Anseong, South Korea

[#]These authors contributed equally to this work.

*** Corresponding Authors**

Jin Won Kim, M.D., Ph.D., F.A.C.C. (Cardiovascular Center, Korea University Guro Hospital, 148, Gurodong-ro, Guro-gu, Seoul 08308, Korea, Tel: 82-2-2626-3021, Fax: 82-2-863-1109, E-mail: kjwmm@korea.ac.kr)

Kyeongsoon Park, Ph.D. (Department of Systems Biotechnology, College of Biotechnology and Natural Resources, Chung-Ang University, Anseong, Gyeonggi-do, 17546, Korea, Tel: 82-31-670-3357. Fax: 82-31-675-1381, E-mail: kspark1223@cau.ac.kr)

Hongki Yoo, Ph.D. (Department of Mechanical Engineering, KAIST, 291 Daehak-ro, Yuseong-gu, Daejeon 305-701, Korea, Tel: 82-42-350-3243, E-mail: h.yoo@kaist.ac.kr)

Supplementary Methods

Synthesis of MAN-PEG-NAC

As targeting ligand molecules, mannose-polyethylene glycol-*N*-acetyl cysteine (MAN-PEG-NAC) was prepared as follows: 300 mg of maleimide-polyethylene glycol 2000-succinimidyl carboxymethyl ester (MAL-PEG-NHS ester; JenKem Technology, Plano, TX, USA), 60 mg of mannosamine (MAN; Sigma Aldrich, St. Louis, MO, USA), and 60 μL of trimethylamine (TEA; Sigma Aldrich) were dissolved in 8 mL of dimethylformamide (DMF; Sigma Aldrich) and reacted overnight. The resulting solution was dialyzed against deionized water using a dialysis membrane (Molecular weight cut-off (MWCO): 1 kDa, Spectrum Laboratories, Inc., Rancho Dominguez, CA, USA) for 2 days to remove excess MAN and freeze-dried to obtain MAN-PEG-MAL. The lyophilized MAN-PEG-MAL (250 mg) was further reacted with NAC (163 mg) in phosphate-buffered saline (PBS; pH 6.9) for 12 h. The reaction mixture was dialyzed against deionized water using a dialysis membrane (MWCO: 1 kDa) for 3 days and lyophilized. The synthesis of MAN-PEG-NAC was confirmed using ^{1}H nuclear magnetic resonance (^{1}H-NMR) spectrometer (DPX 400 MHz, Bruker, Billerica, MA, USA). ^{1}H-NMR (solvent: chloroform-d) peaks were as follows: δ 3.81–4.16 (H from MAN), δ 3.58–3.74 (H of –$C\underline{H_2}C\underline{H_2}O$– from PEG), δ 2.52 (H of MAL-CH_2-$C\underline{H_2}$-CO-PEG), and δ 2.06 ($C\underline{H_3}$ of NAC from MAN-PEG-NAC). The molecular mass of MAN-PEG-NAC was analyzed with a matrix-assisted laser desorption/ionization-time of flight mass spectrometer (Voyager-DETM STR Biospectrometry Workstation, Applied Biosystems, Foster City, CA, USA). The determined molecular mass of MAN-PEG-NAC is approximately 2,690 Da.

Synthesis of MAN-PEG-GC-DOCA-Cy7 (MMR-Cy)

A mannose receptor targetable drug carrier (Mannose (MAN)-polyethylene glycol (PEG)-glycol chitosan (GC)-deoxycholic acid (DOCA)-cyanine 7 (Cy7); MMR-Cy) was fabricated as follows. The COOH of DOCA (100 mg) was pre-activated with 1-ethyl-3-(3-dimethylaminopropyl)-carbodiimide hydrochloride (EDC, 200 mg) and *N*-hydroxysuccinimide (NHS, 125 mg) in methanol (MeOH, 10 mL) for 30 min. Then, pre-activated DOCA was reacted with GC (1 g) dissolved in 300 mL of the co-solvent of deionized water:MeOH (1:2, v/v). After 24 h, the pre-activated COOH of MAN-PEG-NAC (280 mg) with EDC (65 mg) and NHS (40 mg) in MeOH (10 mL) was added to the solution. The mixture was dialyzed against deionized water containing 30% ethanol for 2 days using a dialysis membrane (MWCO: 12-14 kDa, Spectrum Laboratories, Inc.), further dialyzed against deionized water for an additional 2 days, and lyophilized to yield MMR carrier. The synthesis of MMR carrier was confirmed using a ^{1}H-NMR spectrometer (600 MHz NMR; Varian, Palo Alto, CA, USA). ^{1}H-NMR (solvent: deuterium oxide:dimethyl sulfoxide-d_6 = 1:3, v/v) peaks were as follows: δ 0.63-1.82 (H of C$\underline{H}$, C$\underline{H_2}$, and C$\underline{H_3}$ from DOCA), δ 3.3-3.65 (H of –C$\underline{H_2}$C$\underline{H_2}$O– from glycol chitosan and PEG). To obtain MMR-Cy, MMR carrier (1.2 g) was clearly dissolved in 300 mL of the co-solvent of deionized water:MeOH (1:2, v/v), and Cy7-NHS ester (60 mg, Lumiprobe Corporation, Hunt Valley, MD, USA) was then added and further reacted under darkness for additional 24 h. The mixture solution was purified by dialysis in the same manner described above for purification of MMR carrier.

Preparation of theranostic nanodrug (MMR-Lobe-Cy)

Lobeglitazone-loaded MMR-Cy (MMR-Lobe-Cy, termed as a theranostic nanodrug) was prepared as follows: MMR-Cy (1 g) and Lobe (300 mg) were clearly dissolved in 200 mL of the co-solvent of deionized water:MeOH (1:3, v/v) for 3 h, dialyzed against deionized water using a dialysis membrane (MWCO: 6-8 kDa, Spectrum Laboratories, Inc.) for 2 days, and lyophilized for 2 days to yield MMR-Lobe-Cy.

Determination of amount of lobeglitazone and Cy7 within the theranostic nanodrug

To determine drug loading content of lobeglitazone in MMR-Lobe-Cy, MMR-Lobe-Cy (1 mg) was solubilized in 1 mL of acetonitrile:deionized water:formic acid (60:40:0.25, v/v/v), and high-performance liquid chromatography (HPLC; Agilent 1260 series, Agilent Technologies, Santa Clara, CA, USA) was performed [1, 2]. In brief, 5 μL of MMR-Lobe-Cy (1 mg/mL) was eluted at a flow rate of 0.5 mL/min via a mobile phase (acetonitrile:deionized water:formic acid (60:40:0.25, v/v/v)). The calculated amount of lobeglitazone in 1 mg of MMR-Lobe-Cy was 0.258 mg by using the equation obtained from the standard curve of the lobeglitazone [$Y = 19446X + 0.7106$: $R^2 = 1$]. To determine the amount of Cy7, MMR-Lobe-Cy (1 mg) and MMR-Cy (1 mg) were clearly dissolved in dimethyl sulfoxide (DMSO; 1 mL) and the absorbance was measured at 760 nm using a UV-VIS spectrophotometer (NEOGEN, Daejeon, South Korea). The calculated amount of Cy7 in 1 mg of MMR-Lobe-Cy and MMR-Cy was 0.75 μg and 1.6 μg, respectively, by using the following equation obtained from the standard curve of the Cy7 [$Y = 359.95X + 0.0319$ (R^2=0.9994)].

Particle size analysis

For particle size analysis, MMR-Lobe-Cy (1 mg) was dispersed in deionized water (1 mL) and then sonicated for 1 min at 100 W. The particle sizes were determined using a Zetasizer 3000 instrument (Malvern Instruments, Malvern, UK).

Transmission electron microscopy

To determine the shape of the nanoparticle, MMR-Lobe-Cy (0.5 mg) was dispersed in 1 mL of deionized water by vortexing for 1 min, placed on grid, negatively stained with 2 wt% uranyl-acetate solution, and let to dry. The morphology of MMR-Lobe-Cy nanoparticle was observed using an energy filtering transmission electron microscopy (EF-TEM; LEO 912AB OMEGA, Carl Zeiss, Oberkochem, Germany).

In vitro drug release study

To evaluate the release profile of lobeglitazone from MMR-Lobe-Cy, we dispersed the 1 mg of lyophilized MMR-Lobe-Cy in 1 mL of PBS (pH 7.4) by vortexing and sonification (100 W) for 1 min, respectively. Then, the dispersed MMR-Lobe-Cy was introduced into a dialysis membrane (MWCO 6-9 kDa) which was immersed in PBS (20 mL, pH 7.4), and then shaking and oscillating 100 times/min in a water bath (37°C). The amount of lobeglitazone released from MMR-Lobe-Cy was measured by HPLC at predetermined time points, followed by medium replacement with fresh one at each time. We used acetonitrile:H_2O:formic acid (60:40:0.25) as a mobile phase. The analysis was carried out at 0.5 mL/min flow rate with a detection wavelength of 250 nm and the injection volume was arranged as 5 μL.

The stability test of MMR-Lobe-Cy in PBS and 10% FBS-containing DMEM

To test the stability of MMR-Lobe-Cy, 0.5 mg of MMR-Lobe-Cy was added to PBS (pH 7.4) and 10% FBS-containing DMEM (without phenol) and then sonicated at 22.5 W for 1 min using a probe-type sonicator. Then, the hydrodynamic diameters of MMR-Lobe-Cy were measured in two different conditions for 6 days.

Quantitative PCR

Total RNA was extracted from RAW264.7 cells with AccuPrep® Universal RNA Extraction Kit (K-3141; Bioneer, Daejeon, Korea) according to the manufacturer's instructions. One microgram total RNA for each sample was reverse-transcribed using the AccuPower® RocketScript Cycle RT premix (K-2201; Bioneer). The resulting complementary DNA was analyzed by real-time PCR using AccuPower® 2X GreenStar™ qPCR MasterMix (K-6251; Bioneer) under the QuantStudio 6 Flex Real-time PCR system (Thermo Fisher Scientific, Waltham, MA, USA). The cycling conditions were 95℃ for 10 min, followed by 40 cycles of 95℃ for 5 sec, 58℃ for 25 sec and 72℃ for 30 sec. Primers that were used are listed in Table S1. Relative mRNA levels were determined using the comparative Ct method and normalized against GAPDH.

Immunofluorescence

RAW264.7 cells (1×10^5 cells per plate) were seeded in poly L-lysine-coated chamber slides, incubated for 24 h, and then stimulated with LPS and LDL for 24 h. The cells were fixed with 4% paraformaldehyde in PBS for 20 min at room temperature, blocked with 2% bovine serum albumin

for 1 h, and incubated with CD206 antibody (MR5D3; 1:20; Bio-Rad Laboratories, Hercules, CA, USA) overnight at 4℃, followed by incubation with AffiniPure goat anti-rat IgG secondary antibody (405418; 1:50) overnight at 4°C. After washing with PBS, the cells were mounted using a fluorescence mounting medium with DAPI (#E19-18; GBI Labs, Bothell, WA, USA) and examined using a confocal fluorescence microscope (LSM 900, Carl Zeiss, Oberkochen, Germany).

Flow cytometry

RAW264.7 cells were seeded on 60-mm culture dish at a density of 2.5×10^6 cells per dish and incubated for 24 h, followed by stimulation of LPS and LDL for 24 h to induce foam cells. RAW264.7 cells not stimulated with LPS and LDL were used as controls. Density of cells was adjusted to 1×10^7 cells/mL and then 100 μL of cells were transferred to 1.5 mL Eppendorf tube. The cells were incubated with CD206 antibody (MR5D3; 1:10; Bio-Rad Laboratories, CA, USA) for 45 min on ice, washed twice in PBS centrifuging at 300×g for 5 min. Alexa Fluor 488 Goat Anti-Rat IgG secondary antibody (#405418; 1:50; Biolegend, CA, USA) were added, and samples were incubated for 45 min on ice. After washing twice with PBS, 400 μL of PBS were added and the cells were transferred into a new 5 mL round-bottom polystyrene tube. Two control samples were prepared: 1) non-stimulated RAW264.7 cells and foam cells that were not stained with primary and secondary antibody; 2) non-stimulated RAW264.7 cells and foam cells only stained with secondary antibody. Samples were analyzed using an LSRFortessa X-20 cell analyzer (BD Bioscience, San Diego, CA, USA).

Development of Atheromatous Rabbit Model

New Zealand white rabbits (male, 3-month-old; Doo-Yoel Biotech, Seoul, South Korea) served as the atheromatous rabbit model. Atherosclerotic lesions were developed by denuding aortic endothelium with balloon catheter and inducing hypercholesterolemia with high cholesterol diet-feeding (Doo-Yeol Biotech, Seoul, South Korea). New Zealand white rabbits were fed high cholesterol diet (1% cholesterol) for 1 week before balloon injury to induce hypercholesterolemia. 3F Fogarty embolectomy catheter (Edwards Laboratories, Santa Ana, CA, USA) was inserted through carotid artery and balloon denudation was performed at the infrarenal aorta with three pullbacks at a balloon pressure ranging from 0.15 to 0.2 mL to distend the aorta to 1.5-fold of the diameter. Three weeks after balloon injury, rabbits were placed on the 0.1% high cholesterol diet for 6-8 weeks, and normal diet was given with the start of the treatment. After 4 weeks of 1% HCD feeding, the amount of dietary cholesterol supplementation was reduced to 0.1% cholesterol and maintained until the *in vivo* experiments to prevent hepatotoxicity, since the long-term administration of high-cholesterol diet could result in overt fatty liver disease [3].

Serial *in vivo* OCT-NIRF imaging experiments

Total 15 atheromatous rabbit models were randomized into three groups: MMR-Lobe-Cy *versus* oral lobeglitazone *versus* saline control groups. Rabbits in each group were treated with MMR-Lobe-Cy by intravenous injection (10 mg/kg, n = 5), lobeglitazone by oral gavage (2 mg/kg, n = 5), or placebo (saline injection, n = 5), respectively. Rabbits in oral lobeglitazone and control groups were further injected with a targetable NIRF imaging agent, MMR-Cy (4 mg/kg). 24 hours after injection of the aforementioned agents (MMR-Lobe-Cy group: 10 mg/kg of MMR-Lobe-Cy;

oral lobeglitazone group: 2 mg/kg of lobeglitazone and 4 mg/kg of MMR-Cy; saline control group: 4 mg/kg of MMR-Cy), each animal was imaged twice at an interval of 7 days using our customized OCT-NIRF catheter-based imaging strategy [4-6]. Injection doses of MMR-Lobe-Cy and MMR-Cy were compensated based on the amount of Cy7 in each agent and the blood NIRF quantitation by a fluorescence reflectance imaging (FRI) analysis. For baseline OCT-NIRF imaging (designated as Day 0), anesthesia was induced with intravenous injection of 3 mg/kg of alfaxalone (Alfaxan; Jurox, Rutherford, Australia) and maintained with 0.5–2.0% isoflurane (Foran; Choongwae Pharma Co., Seoul, South Korea) in oxygen through an endotracheal tube. The right iliac artery was carefully exposed followed by insertion of a 4 F sheath and then baseline angiography was acquired to evaluate the vessel anatomy. Standard 0.014 inch guide wire was employed through 4 F sheath, and the OCT-NIRF imaging catheter was advanced over the guide wire. OCT-NIRF imaging was performed with a rotation speed of 50 rotation per second, pullback speed of 20 mm/s, pullback length of 80 mm, and pullback direction from proximal to distal aorta under non-occlusive contrast flushing. For follow-up imaging (designated as Day 7), OCT-NIRF catheter was accessed through the left common iliac artery and the same aortic plaque area was imaged with identical settings to analyze the serial changes of plaque features including morphological characteristics and inflammation. After Day 7 follow-up imaging, *ex vivo* and histological validation were performed. The animals were cared for in accordance with the NIH Guide for the Care and Use of Laboratory Animals, and all animal studies were approved by the Institutional Animal Care and Use Committee (IACUC) of Korea University College of Medicine (IACUC approval No. KOREA-2018-0066).

Catheter-based intravascular OCT-NIRF imaging system

We have developed a custom-built catheter-based OCT-NIRF imaging system. Our OCT system obtains high-resolution (axial resolution of ~13 μm) OCT images at an A-scan rate of 117.2 kA-lines/sec and a high frame rate of up to 100 frames/sec using a custom-built high-speed wavelength-swept laser with a repetition rate of 117.2 kHz and a wavelength sweeping range of 110 nm (centered at 1,295 nm) [7]. Excitation of Cy7 dye was done *via* a 730 nm laser diode (WSLP-730-030m-4-B-PD, Wavespectrum Laser Inc., Beijing, China). Optically co-registered data acquisition and spectral separation between OCT, NIRF excitation, and returning NIRF emission were realized by using a dual-mode fiber optic rotary joint (FORJ; MJP-SAPB-131-DC-00954-SA, Princetel, Inc., NJ, USA) in combination with fiber optic components including a custom-built wavelength division multiplexer (WDM; Thorlabs, Inc., NJ, USA) and a double-clad fiber coupler (DCFC; DC1300LEFA, Thorlabs, Inc., NJ, USA). The dual-mode FORJ rotates up to 100 revolutions/sec without non-uniform rotational distortion while maintaining high coupling efficiency with a deviation less than 10%. For helical scanning, the FORJ was mounted onto a motorized stage (T-LSQ-075B, Zaber Technologies, Vancouver, BC, Canada) to be pulled back to a length of 100 mm at a speed of up to 40 mm/sec. In addition, we fabricated a ball lens-based dual-mode imaging catheter for intravascular imaging, with an outer diameter of 0.84 mm, which is comparable to that of the clinically used OCT imaging catheter. The optical power of the OCT and NIRF excitation light were measured to be 35 mW and 13 mW, respectively, at the tip of the imaging catheter. The returning NIRF emission light was recorded synchronously to the wavelength-swept laser, so the NIRF acquisition rate is equal to the A-scan rate of the OCT system. For real-time integrated OCT-NIRF image visualization in the operating room environment, both

recorded OCT and NIRF emission light were processed by multithreading programming techniques.

Distance calibration and normalization of NIRF emission intensity

Since the NIRF emission intensity attenuates as a function of the distance from the imaging catheter to the arterial wall [8], a calibration algorithm for the attenuated intensity is required for accurate and undistorted inflammation assessment based on NIRF data. Details for the calibration algorithm has been described in our previous publications [4, 5]. Briefly, first, OCT-NIRF imaging was performed on a fluorescent phantom tube filled with homogeneous Cy7 milk solution to obtain the relationship between the NIRF emission intensity and the distance. Second, the distance between the imaging catheter and the surface of the phantom tube was estimated in all OCT images of the pullback by using the automated lumen segmentation algorithm [9]. Third, the pairs between the NIRF emission intensity and the corresponding distance were approximated by a two-term exponential fitting function:

$$f(x) = (a * \exp(-b * x) + c * \exp(-d * x))/(a + c),$$

where a, b, c, and d denote fitting coefficients, x denotes the distance and $f(x)$ denotes the expected attenuation ratio at the distance of x. Here, we introduced the calibration function, $comp(x) = 1/f(x)$, the reciprocal of the exponential fitting function. Thus, by using each NIRF emission intensity and the corresponding calibration function value, we can identify the calibrated NIRF emission intensity data obtained from rabbit aorta.

Despite the calibration for the attenuation along the distance, the NIRF emission intensity may still be affected by other factors, such as variations of the imaging system (imaging catheter, laser

diode output, and detector sensitivity) and differences in pharmaco-kinetics and -dynamics between individual rabbits. In order to exclude the potential distortion of the NIRF emission intensity due to the factors mentioned above, we normalized the NIRF emission intensity to the plaque target-to-background ratio (pTBR):

$$pTBR(y_{calib}) = \frac{y_{calib}}{\overline{y_{calib}}},$$

where y_{calib} and $\overline{y_{calib}}$ denote the distance-calibrated NIRF emission intensity and its background value, respectively, and $pTBR(\cdot)$ denotes the operators for pTBR calculation. The background value was determined by averaging the five lowest NIRF emission intensities in neighboring normal-looking intact segments. On the graphical user interface for OCT-NIRF image acquisition, the entire calibration and normalization processes were automatically performed as post-processing.

Picro-Sirius Red staining

For Picro-Sirius Red (PSR) Staining, the sections were hydrated to distilled water and placed in phosphomolybdic acid solution (0.2%) for 10 sec, followed by dipping slide in distilled water. Next, the slides were incubated with PSR solutions for 1h, and then rinsed quickly two times in acetic acid solution (0.5%). The sections were dehydrated, cleared and mounted in synthetic resin.

Detailed methods for quantifying Oil-red-O and Picro-Sirius Red stained images

Oil-red O (ORO) and/or PSR-stained tissue images were properly adjusted by white balance. Second, to separate the red positive-stained pixels from both ORO and PSR staining images, the default red-green-blue (RGB) color-space was converted to hue-saturation-value (HSV) color-space. Third, Gaussian filtering with a sigma of 20 pixels, binarization by thresholding and image erosion by a structuring element with a radius of 25 pixels were sequentially performed on the value channel for the segmentation of the tissue section areas. Note that since background of the images obtained by brightfield microscopy was white, the value channel was inverted to set the background as zero before the following image processing. Fourth, after applying Gaussian filtering with a sigma of 20 pixels to the saturation channel, red positive-stained pixels in the pre-determined tissue section areas were chosen according to the corresponding hue ($-50^{o} \sim 50^{o}$) and saturation (>6%) channels. Finally, the quantified positive-stained ratio was calculated by dividing the number of pixels in the segmented tissue section area by that in the positive-stained area. The parameters introduced above have been determined empirically and can be adjusted slightly depending on the type of the histological staining. This quantitation processes were implemented using ImageJ (National Institute of Health, Bethesda, MD, USA) and MATLAB software (R2017a; The MathWorks, Natick, MA, USA).

***In vitro* cytotoxicity**

The cytotoxicity of MMR-Lobe-Cy was evaluated using cell counting kit-8 (CCK-8, Dojindo Laboratories, Japan). RAW264.7 cells were seeded at 5×10^{5} cells per well in a 96-well plate, and allowed to adhere for 24 h. The cells were treated with LPS (200 ng/mL) in the presence of LDL

(100 μg/mL) for 24 h to induce foam cells. Cells treated with PBS were prepared as controls. Then, cells were maintained in a serum-free medium containing various concentrations of MMR-Lobe-Cy containing 0, 1, 5, 10, 20, 50 μM of lobeglitazone for 24 h. After washing with PBS, 10 μL of CCK-8 solution was added to each well with 100 μL of medium, and the cells were further incubated for 2 h. The optical density values were read using a microplate reader at a wavelength of 450 nm. All experiments were conducted in triplicate. Cell viability was defined as the percentage of living cells per that of cells at 0 μM.

Complete blood count and biochemistry

Blood was collected in ethylenediaminetetraacetic acid (EDTA) tube, and then hematological parameters such red blood cells, hemoglobin, platelets, and white blood cells were analyzed using an automated hematology system (XN-9100; Sysmex Corporation, Kobe, Japan). To measure the biochemistry profile, blood was sampled in serum separator blood collection tube, left to clot for at least 30 min, and subsequently centrifuged at 3000 rpm for 5 min. The serum was aliquoted and stored at -80°C until measured on an automatic analyzer (DRI-CHEM NX500i; FUJIFLIM, Japan). The samples were assayed for aspartate transaminase (AST), alanine transaminase (ALT), blood urea nitrogen (BUN), and creatinine (CRE).

Table S1. Primer sequence for qPCR.

Gene	Forward Primer 5'-3'	Reverse Primer 5'-3'
Mrc1	CAACCAAAGCTGACCAAAGG	CCGGCACCTATCACAATCAG
GAPDH	GAACGGATTTGGCCGTATTG	GTTGAATTTGCCGTGAGTGG

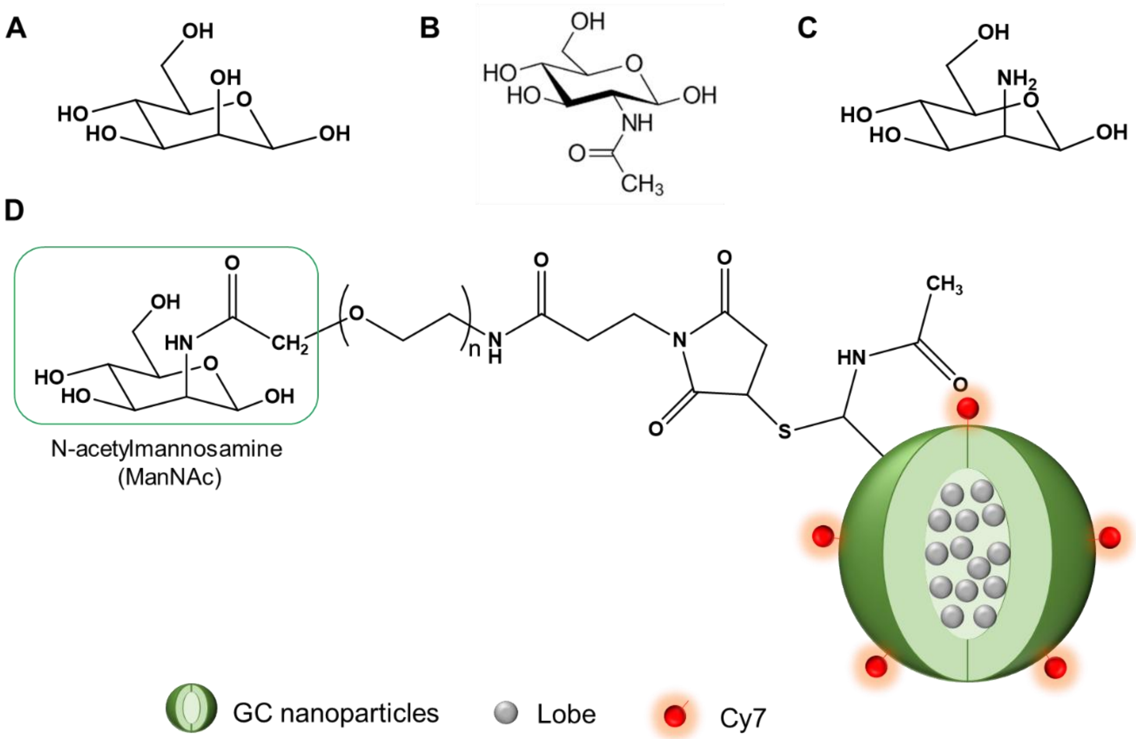

Figure S1. Chemical structures and the major chair forms of (A) D-mannose, (B) N-acetylglucosamine (GlcNAc), and (C) D-mannosamine. (D) Chemical structure of MMR-Lobe-Cy. N-acetylmannosamine moiety (green box) in MMR-Lobe-Cy had similar structure with mannose.

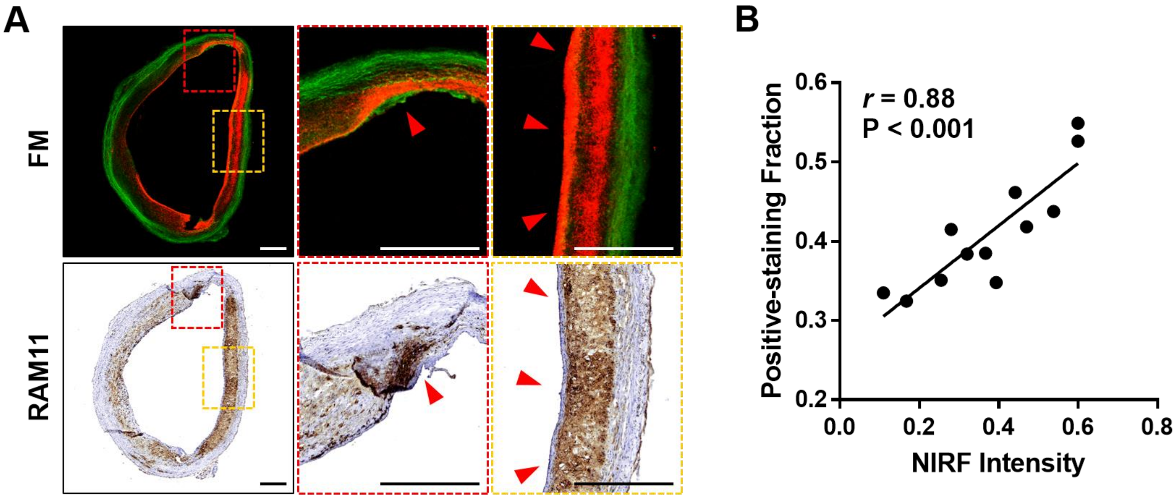

Figure S2. Co-localization of MMR-Lobe-Cy and macrophages in atherosclerotic plaque. (A) Representative FM image and the corresponding RAM11 immunohistochemistry of a sectioned plaque sample. (B) Correlation plot between the averaged NIRF intensity and positive-staining fraction.

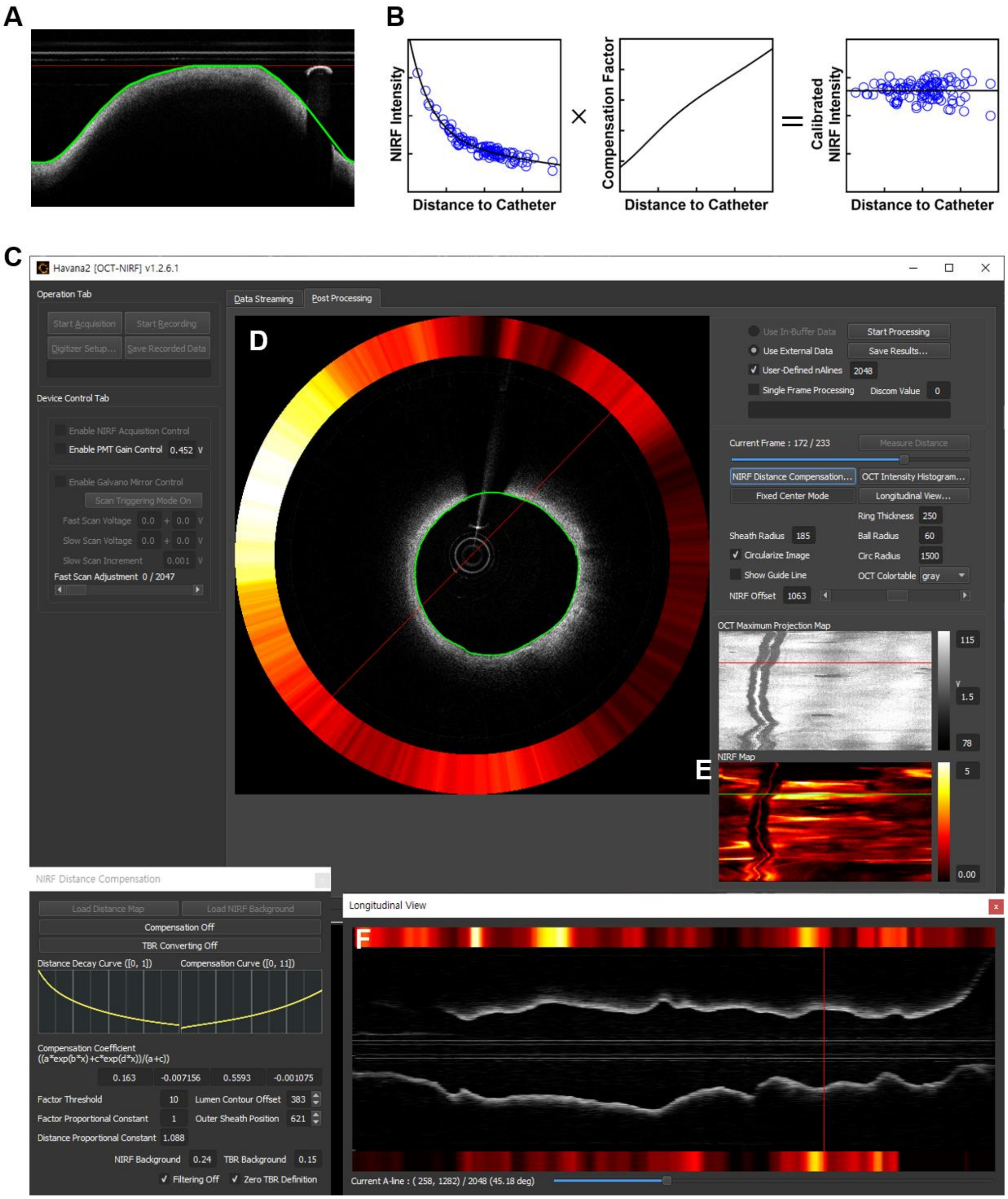

Figure S3. Customized single catheter-based OCT-NIRF imaging system and automatic image processing for OCT-NIRF visualization. (A) Automated lumen contour detection, and (B) NIRF intensity distance calibration. (C) User friendly interface allowing real-time display of distance calibrated OCT-NIRF images. (D) Corrected OCT-NIRF cross sectional image. (E) *In vivo* NIRF 2-dimensional mapping. (F) Longitudinal OCT-NIRF cross-section image.

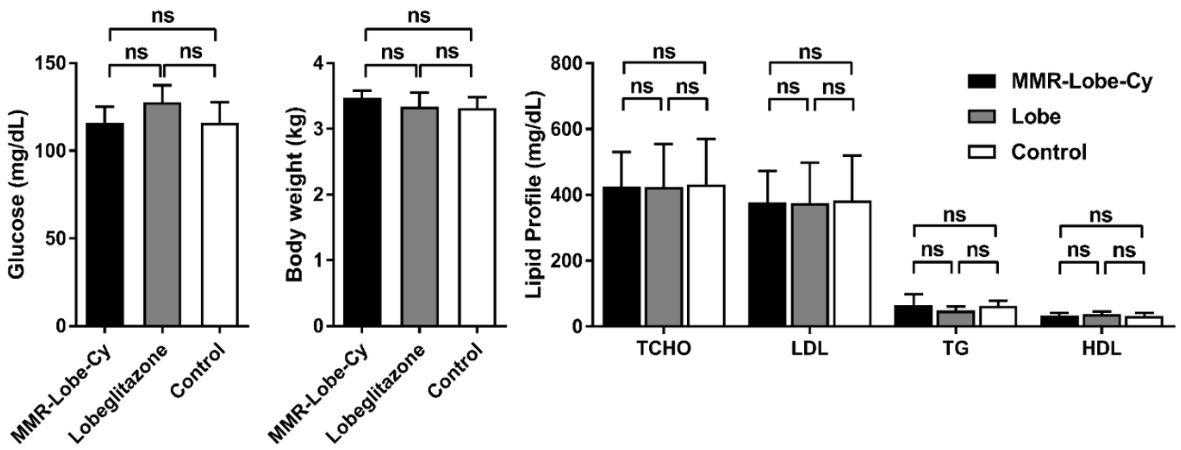

Figure S4. Evaluation of metabolic parameters after treatment of MMR-Lobe-Cy, lobeglitaonze or saline. Comparison of glucose, body weight, and serum lipid levels of rabbits treated with MMR-Lobe-Cy, lobeglitazone, and saline. TCHO, serum total cholesterol; LDL, low-density lipoprotein; TG, triglycerides; HDL, high-density lipoprotein.

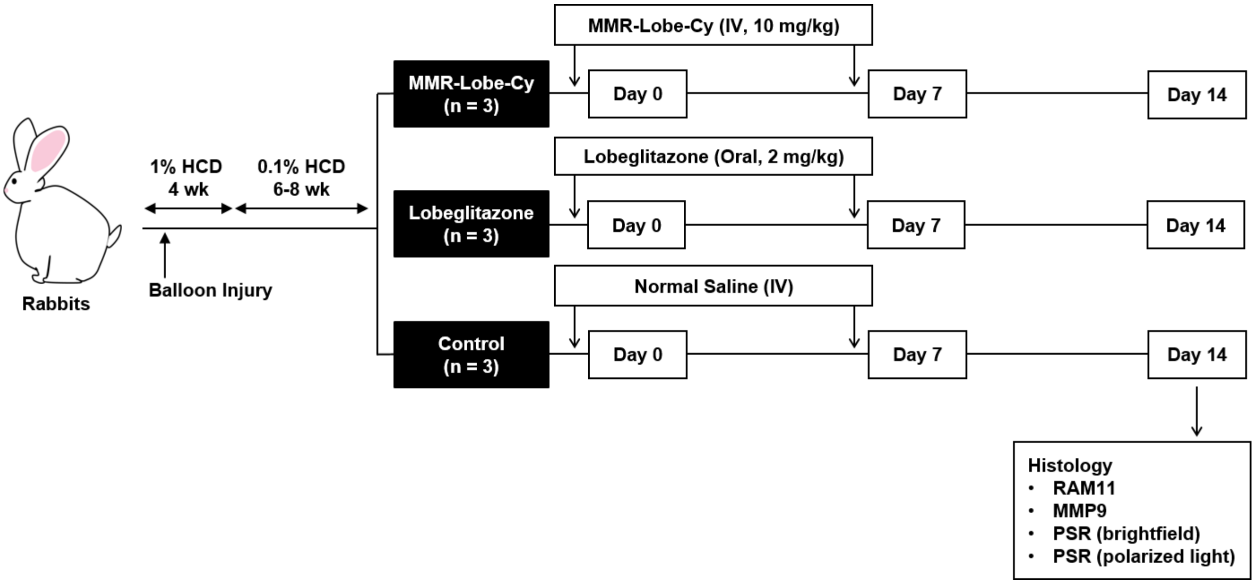

Figure S5. Schematic diagram of *in vivo* assessment of macrophage contents, protease expression, and collagen contents after MMR-Lobe-Cy treatment at Day 14. Rabbit atherosclerotic model was developed by high cholesterol diet feeding and balloon injury. Rabbits were injected with MMR-Lobe-Cy (n = 3), lobeglitazone (n = 3) or saline (n = 3) twice with a one week interval, and then euthanized at 7 days after final injection of agents for histological validation (Day 14).

Movie S1. (related to Figure 3) *In vivo* serial OCT-NIRF imaging of the inflamed plaque in MMR-Lobe-Cy group. Strong NIRF signals were detected in OCT-NIRF images on Day 0 (left) and significantly decreased in follow-up images on Day 7 (right).

Movie S2. (related to Figure 3) Day 0 (left) and Day 7 (right) *in vivo* serial OCT-NIRF imaging of the inflamed plaque in oral lobeglitazone group. MMR-Cy were used as a signal enhancer.

Movie S3. (related to Figure 3) Day 0 (left) and Day 7 (right) *in vivo* serial OCT-NIRF imaging of the inflamed plaque in saline control. The inflammatory NIRF signals in the plaque even increased over time. MMR-Cy were used as a signal enhancer.

References

1. Choi JY, Ryu J, Kim HJ, Song JW, Jeon JH, Lee DH, et al. Therapeutic Effects of Targeted PPAR Activation on Inflamed High-Risk Plaques Assessed by Serial Optical Imaging In Vivo. Theranostics. 2018; 8: 45-60.
2. Park S, Ahn JW, Jo Y, Kang HY, Kim HJ, Cheon Y, et al. Label-Free Tomographic Imaging of Lipid Droplets in Foam Cells for Machine-Learning-Assisted Therapeutic Evaluation of Targeted Nanodrugs. ACS nano. 2020; 14: 1856-65.
3. Kolodgie FD, Katocs AS, Jr., Largis EE, Wrenn SM, Cornhill JF, Herderick EE, et al. Hypercholesterolemia in the rabbit induced by feeding graded amounts of low-level cholesterol. Methodological considerations regarding individual variability in response to dietary cholesterol and development of lesion type. Arterioscler Thromb Vasc Biol. 1996; 16: 1454-64.
4. Kim S, Lee MW, Kim TS, Song JW, Nam HS, Cho HS, et al. Intracoronary dual-modal optical coherence tomography-near-infrared fluorescence structural-molecular imaging with a clinical dose of indocyanine green for the assessment of high-risk plaques and stent-associated inflammation in a beating coronary artery. Eur Heart J. 2016; 37: 2833-44.
5. Lee S, Lee MW, Cho HS, Song JW, Nam HS, Oh DJ, et al. Fully integrated high-speed intravascular optical coherence tomography/near-infrared fluorescence structural/molecular imaging in vivo using a clinically available near-infrared fluorescence-emitting indocyanine green to detect inflamed lipid-rich atheromata in coronary-sized vessels. Circ Cardiovasc Interv. 2014; 7: 560-9.
6. Yoo H, Kim JW, Shishkov M, Namati E, Morse T, Shubochkin R, et al. Intra-arterial catheter for simultaneous microstructural and molecular imaging in vivo. Nature medicine. 2011; 17: 1680-4.
7. Cho HS, Jang SJ, Kim K, Dan-Chin-Yu AV, Shishkov M, Bouma BE, et al. High frame-rate intravascular optical frequency-domain imaging in vivo. Biomedical Optics Express. 2014; 5: 223-32.
8. Ughi GJ, Verjans J, Fard AM, Wang H, Osborn E, Hara T, et al. Dual modality intravascular optical coherence tomography (OCT) and near-infrared fluorescence (NIRF) imaging: a fully automated algorithm for the distance-calibration of NIRF signal intensity for quantitative molecular imaging. The international journal of cardiovascular imaging. 2015; 31: 259-68.
9. Nam HS, Kim CS, Lee JJ, Song JW, Kim JW, Yoo H. Automated detection of vessel lumen and stent struts in intravascular optical coherence tomography to evaluate stent apposition and neointimal coverage. Med Phys. 2016; 43: 1662.
